# Quantitative Contribution of IL2Rγ to the Dynamic Formation of IL2-IL2R Complexes

**DOI:** 10.1371/journal.pone.0155684

**Published:** 2016-05-19

**Authors:** Luis F. Ponce, Karina García-Martínez, Kalet León

**Affiliations:** Center of Molecular Immunology, System Biology Department, Habana, 11600, Cuba; Technische Universität Dresden, GERMANY

## Abstract

Interleukin-2 (IL2) is a growth factor for several immune cells and its function depends on its binding to IL2Rs in the cell membrane. The most accepted model for the assembling of IL2-IL2R complexes in the cell membrane is the *Affinity Conversion Model* (ACM). This model postulates that IL2R receptor association is sequential and dependent on ligand binding. Most likely free IL2 binds first to IL2Rα, and then this complex binds to IL2Rβ, and finally to IL2Rγ (γc). However, in previous mathematical models representing this process, the binding of γc has not been taken into account. In this work, the quantitative contribution of the number of IL2Rγ chain to the IL2-IL2R apparent binding affinity and signaling is studied. A mathematical model of the affinity conversion process including the γ chain in the dynamic, has been formulated. The model was calibrated by fitting it to experimental data, specifically, Scatchard plots obtained using human cell lines. This paper demonstrates how the model correctly explains available experimental observations. It was estimated, for the first time, the value of the kinetic coefficients of IL2-IL2R complexes interaction in the cell membrane. Moreover, the number of IL2R components in different cell lines was also estimated. It was obtained a variable distribution in the number of IL2R components depending on the cell type and the activation state. Of most significance, the study predicts that not only the number of IL2Rα and IL2Rβ, but also the number of γc determine the capacity of the cell to capture and retain IL2 in signalling complexes. Moreover, it is also showed that different cells might use different pathways to bind IL2 as consequence of its IL2R components distribution in the membrane.

## Introduction

Interleukin-2 (IL2) is a protein initially identified as a T cell growth factor [1]. IL2 is mainly produced by activated CD4+CD25- (helper) T cells, and induces the proliferation of these and others cells like CD8+ T cells, B and NK cells [2]. For this reason, it has been used to treat immune-deficiencies like HIV, and induce immune response against tumors [3]. Nevertheless, it has been shown that IL2 also acts as the main growth factor of CD4+CD25+ Regulatory T cells [2]. The immunosuppressive properties of this type of cells, has led to discussions about the actual role and suitability of IL2 in the treatment of the above-mentioned therapies [4,5].

Interleukin 2 mediates its functions in the target cell through the interleukin 2 receptor (IL2R). IL2R is a multimeric functional protein consisting of three different chains: IL2Rα [6], IL2Rβ [7] and γc [8]. The first two components are able to interact with IL2 with *K*_*d*_~10^-8^M [9] and *K*_*d*_~10^-7^M [10] respectively. On the other hand, the third component (γc) is unable to interact with IL2 alone [11]. Simultaneous interaction between IL2 and multiple components of IL2R in the cell membrane leads to an increase of IL2 capture and retention by cells, which is interpreted as other IL2R conformations with different apparent affinity. The apparent affinity depends on the interacting components of IL2R: the high affinity receptor IL2Rα-IL2Rβ-γc (*K*_*d*_~10^-11^M) [12], the pseudo-high affinity receptor IL2Rα-IL2Rβ (*K*_*d*_~10^-10^M) [13] and the intermediate affinity receptor IL2Rβ-γc (*K*_*d*_~10^-9^M) [14]. The signaling pathway is activated after the heterodimerization of IL2Rβ and γc, mediated by IL2 [15].

IL2 activity in different cell types is given by the differential distribution of the IL2R chains on the cell membrane. The distribution of IL2Rα in different cells has been studied by several techniques, including Scatchard method. These studies have revealed that the expression of IL2Rα highly increases during the activation of T cells. For this reason, IL2Rα expression has been established as a marker of activated T cells. On the other hand, IL2Rβ is highly expressed in NK and CD8+ T cells. The differences on IL2Rα and IL2Rβ expression leads to differences in the capacity of cells to capture and retain IL2 [9,12,14,16]. It also, explains the differences in the sensitivity of different cells to IL2 stimulation [17].

The γc was the last component of IL2R to be discovered [18]. Now it is known that γc is part or the functional receptors for other cytokines like IL4, IL7, IL9, IL15 and IL21 [19]. The interaction between γc and IL2 only occurs after the previous association of the ligand with IL2Rβ [8,11]. Therefore, the contribution of γc to the apparent affinity of IL2R species is not obvious. Additionally, the distribution of γc in different cells and its influence on the cell sensitivity to IL2 variations has been less studied.

The proportion of the γc expressed on the cell membrane, in relation to IL2Rβ or other cytokine receptors, might determine the competition for using this common chain by different cytokines. There are two possibilities: a) γc is in excess with respect to IL2Rβ and other cytokines receptors ensuring its accessibility to all cytokine receptors; or b) γc is in defect regulating its availability to different cytokines receptors and the overlap of different signaling cascades in the cell.

The assembling mechanism of IL2-IL2R complexes on the cell membrane remains to be elucidated. The most accepted model is the *Affinity Conversion Model* (ACM). It postulates that IL2R association is sequential and dependent on ligand binding [20]. This is: IL2Rα, IL2Rβ and γc remain separates in the absence of IL2 at the cell membrane, and they only heterodimerize after ligand binding. Initially free IL2 binds to IL2Rα, and then this complex binds to IL2Rβ, and finally γc follows. Although several mathematical formulations of this model the binding of γc is commonly not taken into account in the dynamic of IL2-IL2R association [17,21]. Recent experimental observations support the ACM. The crystal of high affinity IL2R-IL2 complex shows no contact between the extracellular domains of IL2Rα and either IL2Rβ or γc [22,23]. Moreover, Rickert [11] did not detect interaction between IL2Rβ and γc in the absence of IL2, despite the large interphase between these chains in the above-mentioned complex. However, other experimental results suggest a more complex dynamic of IL2-IL2R assembling. FRET technique studies have indicated that IL2Rα and IL2Rβ [24], and IL2Rβ and γc [25], are close enough to interact in the cell membrane in absence of IL2, preforming some type of dimers.

In this work, it is studied how γc chain quantitatively contributes to the IL2-IL2R apparent binding affinity and signaling. This matter passes through the understanding of the IL2-IL2R assembling mechanism. Taking all that into account a mathematical model was formulated including the minimal biology known for the IL2-IL2R system. It is considered the existence of three different chains that coexist in the cell membrane and dynamically interact depending on previous ligand binding. The model we proposed is based in the affinity conversion model for the IL2-IL2R assembling and explicitly includes the γc in the dynamic.

It can be observed in a further reading, that the model properly explains the available experimental observations. It was estimated, for the first time, the kinetic coefficients of IL2-IL2R complexes interaction in the cell membrane. Moreover, the number of IL2R components in different cell lines is also forecasted. It was obtained a variable distribution in the number of IL2R components depending on the cell type and the activation state. It is shown that the IL2Rs expression determines how different cells might use different pathway of IL2-IL2R assembling. Of most significance this study predicts that not only the number of IL2Rα and IL2Rβ, but also the number of γc determine the capacity of the cell to capture and retain IL2 in signalling complexes.

## Methods

### Mathematical Model

**Fig 1:**
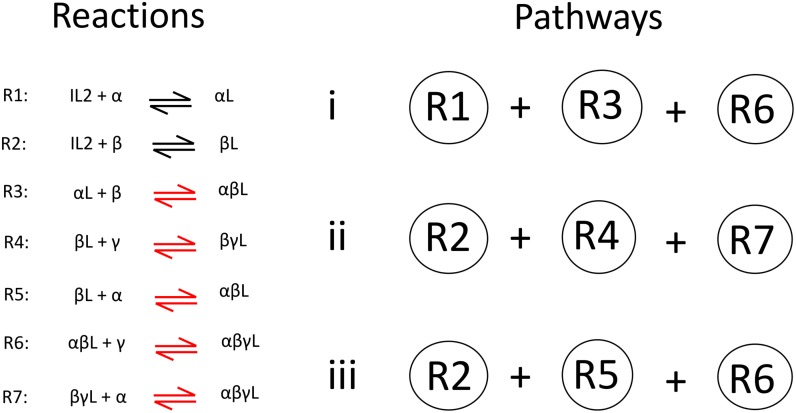
Scheme for the assembly of high affinity IL2 receptor (IL2-IL2Rα-IL2Rβ-γc). Left panel shows the reactions considered in the model network. The red color indicates that the kinetic coefficients of the reactions are unknown parameters. Right panel shows the reactions conforming each pathway of IL2-IL2R assembling mechanism.

The presented model considers that IL2R components interact sequentially, upon IL2 binding to the cell. Furthermore, differently from previous works, it explicitly includes the binding of γ chain in the IL2-IL2R complex assembling. Three different pathways (see Fig 1) are included in the model: (i) free IL2 is captured by IL2Rα forming a dimer, the resulting complex binds to L2Rβ and finally to γc; (ii)-(iii) free IL2 is captured by IL2Rβ, and the complex IL2-IL2Rβ binds first to (ii) IL2Rα and then to γc, or to (iii) γc and then to IL2Rα.

The following 9 ordinary differential equations and an algebraic relation represent these processes. In Tables 1–3 are summarized the definitions of necessary variables and parameters.

$$\frac{dN_{\alpha L}^{i}}{dt}=k_{\alpha L}N_{\alpha }^{i}{\left[L\right]}^{i}-k_{-\alpha L}N_{\alpha L}^{i}-\frac{k_{\alpha L\beta }}{A^{i}}N_{\alpha L}^{i}N_{\beta }^{i}+k_{-\alpha L\beta }N_{\alpha \beta L}^{i}-k_{\text{int}}N_{\alpha L}^{i}$$ (1)

$$\frac{dN_{\beta L}^{i}}{dt}=k_{\beta L}N_{\beta }^{i}{\left[L\right]}^{i}-k_{-\beta L}N_{\beta L}^{i}-\frac{k_{\beta L\alpha }}{A^{i}}N_{\beta L}^{i}N_{\alpha }^{i}+k_{-\beta L\alpha }N_{\alpha \beta L}^{i}-\frac{k_{\beta L\gamma }}{A^{i}}N_{\beta L}^{i}N_{\gamma }^{i}+k_{-\beta L\gamma }N_{\beta \gamma L}^{i}-k_{\text{int}}N_{\beta L}^{i}$$ (2)

$$\frac{dN_{\alpha \beta L}^{i}}{dt}=\frac{k_{\alpha L\beta }}{A^{i}}N_{\alpha L}^{i}N_{\beta }^{i}-k_{-\alpha L\beta }N_{\alpha \beta L}^{i}+\frac{k_{\beta L\alpha }}{A^{i}}N_{\beta L}^{i}N_{\alpha }^{i}-k_{-\beta L\alpha }N_{\alpha \beta L}^{i}-\frac{k_{\alpha \beta L\gamma }}{A^{i}}N_{\alpha \beta L}^{i}N_{\gamma }^{i}+k_{-\alpha \beta L\gamma }N_{\alpha \beta \gamma L}^{i}-k_{\text{int}}N_{\alpha \beta L}^{i}$$ (3)

$$\frac{dN_{\beta \gamma L}^{i}}{dt}=\frac{k_{\beta L\gamma }}{A^{i}}N_{\beta L}^{i}N_{\gamma }^{i}-k_{-\beta L\gamma }N_{\beta \gamma L}^{i}-\frac{k_{\beta \gamma L\alpha }}{A^{i}}N_{\beta \gamma L}^{i}N_{\alpha }^{i}+k_{-\beta \gamma L\alpha }N_{\alpha \beta \gamma L}^{i}-k_{\text{int}}^{\text{sig}}N_{\beta \gamma L}^{i}$$ (4)

$$\frac{dN_{\alpha \beta \gamma L}^{i}}{dt}=\frac{k_{\alpha \beta L\gamma }}{A^{i}}N_{\alpha \beta L}^{i}N_{\gamma }^{i}-k_{-\alpha \beta L\gamma }N_{\alpha \beta \gamma L}^{i}+\frac{k_{\beta \gamma L\alpha }}{A^{i}}N_{\beta \gamma L}^{i}N_{\alpha }^{i}-k_{\beta \gamma L\alpha }N_{\alpha \beta \gamma L}^{i}-k_{\text{int}}^{\text{sig}}N_{\alpha \beta \gamma L}^{i}$$ (5)

$$\frac{dN_{\alpha }^{i}}{dt}=N_{\alpha 0}^{i}k_{\text{int}}-k_{\text{int}}N_{\alpha }^{i}-k_{\alpha L}N_{\alpha }^{i}{\left[L\right]}^{i}+k_{-\alpha L}N_{\alpha L}^{i}-\frac{k_{\beta L\alpha }}{A^{i}}N_{\beta L}^{i}N_{\alpha }^{i}+k_{-\beta L\alpha }N_{\alpha \beta L}^{i}-\frac{k_{\beta \gamma L\alpha }}{A^{i}}N_{\beta \gamma L}^{i}N_{\alpha }^{i}+k_{-\beta \gamma L\alpha }N_{\alpha \beta \gamma L}^{i}$$ (6)

$$\frac{dN_{\beta }^{i}}{dt}=N_{\beta 0}^{i}k_{\text{int}}-k_{\text{int}}N_{\beta }^{i}-k_{\beta L}N_{\beta }^{i}{\left[L\right]}^{i}+k_{-\beta L}N_{\beta L}^{i}-\frac{k_{\alpha L\beta }}{A^{i}}N_{\alpha L}^{i}N_{\beta }^{i}+k_{-\alpha L\beta }N_{\alpha \beta L}^{i}$$ (7)

$$\frac{dN_{\gamma }^{i}}{dt}=N_{\gamma 0}^{i}k_{\text{int}}-k_{\text{int}}N_{\gamma }^{i}-\frac{k_{\beta L\gamma }}{A^{i}}N_{\beta L}^{i}N_{\gamma }^{i}+k_{-\beta L\gamma }N_{\beta \gamma L}^{i}-\frac{k_{\alpha \beta L\gamma }}{A^{i}}N_{\alpha \beta L}^{i}N_{\gamma }^{i}+k_{-\alpha \beta L\gamma }N_{\alpha \beta \gamma L}^{i}$$ (8)

$$\frac{dN_{\text{int}}^{i}}{dt}=k_{\text{int}}\left(N_{\alpha L}^{i}+N_{\alpha \beta L}^{i}+N_{\beta L}^{i}\right)+k_{\text{int}}^{\text{sig}}\left(N_{\beta \gamma L}^{i}+N_{\alpha \beta \gamma L}^{i}\right)$$ (9)

$${\left[L\right]}^{i}={\left[L\right]}_{0}^{i}-\frac{N_{\text{cells}}}{N_{A}V}\left(N_{\alpha L}^{i}+N_{\beta L}^{i}+N_{\alpha \beta L}^{i}+N_{\beta \gamma L}^{i}+N_{\alpha \beta \gamma L}^{i}+N_{\text{int}}^{i}\right)$$ (10)

**Table 1:** Definition of model variables.

Variable	Definition
[*L*]^*i*^	Free ligand concentration in solution containing cells from i-th cell type
$N_{\text{int}}^{i}$	Total number of internalized ligands in the i-th cell type
$N_{\alpha }^{i},N_{\beta }^{i},N_{\gamma }^{i}$	Total number of free IL2Rα, IL2Rβ and γc on the membrane of the i-th cell type
$N_{\alpha L}^{i},N_{\beta L}^{i}$	Number of IL2-IL2Rα and IL2-IL2Rβ dimers on the membrane of the i-th cell type
$N_{\alpha \beta L}^{i},N_{\beta \gamma L}^{i},N_{\alpha \beta \gamma L}^{i}$	Number of IL2-IL2Rα-IL2Rβ, IL2-IL2Rβ-γc, IL2-IL2Rα-IL2Rβ-γc complexes on the membrane of the i-th cell type

**Table 2:** Model parameters, with values taken from literature.

Parameter	Definition	Selected value	References
$k_{\alpha L}^{*}$	Association rate between IL2Rα and soluble IL2	7.8 × 10^6^ M^−1^s^−1^	[26]
*k*_−*αL*_	Dissociation rate of IL2 from IL2Rα	0.4 s^−1^*	[26]
*k*_*βL*_	Association rate between IL2Rβ and soluble IL2	7.9 × 10^5^ M^−1^s^−1^	[27]
*k*_*−βL*_	Dissociation rate of IL2 from IL2Rβ	0.22 s^−1^	[27]
*k*_int_	Constitutive internalization rate of IL2R components	7.0 × 10^−3^ min^−1^	[28]
$k_{\text{int}}^{\text{sig}}$	Ligand induced internalization rate of signaling IL2R components	4.0 × 10^−2^ min^−1^	[28]

* the value of *k*_*-αl*_ were slightly adjusted in order to be in agree with the low affinity phases of Scatchard plots of HUT and PHA activated blast cells.

**Table 3:** Model parameters, whose values are estimated by fitting to data.

Parameter	Definition	Range used in model fitting
*k*_*αLβ*_	Association rate of IL2Rβ to IL2-IL2Rα complex on the cell membrane	(5.0 × 10^−6^ − 1.5×10^−2^)*s*^−1^
*k*_*βLα*_ (*k*_*βγLα*_)	Association rate of IL2Rα to IL2-IL2Rβ-γc (IL2-IL2Rα-IL2Rβ-γc) complex on the cell membrane	(5.0 × 10^−6^ − 1.5×10^−2^)*s*^−1^
*k*_*βLγ*_ (*k*_*αβLγ*_)	Association rate of γc to IL2-IL2Rβ (IL2-IL2Rα-IL2Rβ) complex on the cell membrane	(5.0 × 10^−6^ − 1.5×10^−2^)*s*^−1^
*k*_*−αLβ*_	Dissociation rate of IL2Rβ from IL2-IL2Rα-IL2Rβ complex	(1.7 × 10^−3^ − 0.22)*s*^−1^
*k*_*−βLα*_ (*k*_*−βγLα*_)	Dissociation rate of IL2Rα from IL2-IL2Rα-IL2Rβ (IL2-IL2Rα-IL2Rβ-γc) complex	(1.7 × 10^−3^ − 0.60)*s*^−1^
*k*_*−βLγ*_ (*k*_*−αβLγ*_)	Dissociation rate of γc from IL2-IL2Rβ-γc (IL2-IL2Rα-IL2Rβ-γc) complex	(1.7 × 10^−3^ − 4.0)*s*^−1^
*N*_*α*0_	Total number of IL2Rα at the beginning of the binding assay	40000 − 80000 ^A1^
		20000 − 30000 ^A2^
		2000 − 30000 ^A3^
		10000 − 60000 ^A4^
		200 − 2000 ^B1^
		10 − 400 ^B2^
		7000 − 10000 ^B3^
		100 − 500^B4^
*N*_*β*0_	Total number of IL2Rβ at the beginning of the binding assay	5000 − 10000^A1^
		2000 − 6000 ^A2^
		500 − 6000 ^A3^
		1500 − 2500^A4^
		10000 − 150000 ^B1^
		20000 − 50000^B2^
		20000 − 170000^B3^
		100 − 30000^B4^
*N*_*γ*0_	Total number of γc at the beginning of the binding assay	2000 − 100000 ^A1^
		100 − 10000 ^A2^
		150 − 6000^A3^
		150 − 9000^A4^
		10000 − 150000^B1^
		30000 − 300000 ^B2^
		12000 − 170000 ^B3^
		100 − 30000 ^B4^

The superindexes in the ranges for the number of IL2Rs at the beginning of the experiment refers to the corresponding cells types A1-A4 and B1-B4.

Eqs 1 and 2 correspond to the dynamics of IL2-IL2Rα and IL2-IL2Rβ complexes respectively. The first and second terms of eq 1 (2) correspond to the association and dissociation processes of free IL2 to IL2Rα (IL2Rβ). The third and fourth terms of eq 1 (2) correspond to the association of IL2Rβ to the IL2-IL2Rα complex (IL2Rα to IL2-IL2Rβ complex) on the cells membrane and the dissociation of IL2Rβ (IL2Rα) from IL2-IL2Rα-IL2Rβ complex, respectively. The fifth and sixth terms in eq 2 are related to the formation and dissociation of IL2-IL2Rβ-γc, due to the interaction between IL2-IL2Rβ complex and γc.

(Eq 3) describes the dynamic of IL2-IL2Rα-IL2Rβ complexes. The first two terms correspond to the formation and dissociation of the IL2-IL2Rα-IL2Rβ complex, by the interaction of IL2Rβ with IL2-IL2Rα complex. The third and fourth terms correspond to formation and dissociation of IL2-IL2Rα-IL2Rβ complex by the interaction between IL2Rα and IL2-IL2Rβ complex. The fifth and sixth terms correspond to the association and dissociation of γc to the IL2-IL2Rα-IL2Rβ complex.

(Eq 4) describes the dynamic of IL2-IL2Rβ-γc complexes. The first two terms are related to processes of association and dissociation of γc with the dimer IL2-IL2Rβ. The third and fourth terms correspond respectively to the interaction of IL2Rα and IL2-IL2Rβ-γc.

(Eq 5) describes the dynamics of high affinity IL2-IL2Rα-IL2Rβ-γc complexes. The first two terms correspond to the formation and dissociation of high affinity complex by the interaction of γc and IL2-IL2Rα-IL2Rβ complex. The third and fourth terms correspond to the interaction of IL2Rα with IL2-IL2Rβ-γc complex.

Last term of eqs (1–5) correspond to the process of internalization of IL2-IL2R complexes. We discriminate between the constitutive internalization (described by parameter *k*_int_) and the internalization of signalling complexes (described by parameter $k_{\text{int}}^{\text{sig}}$).

Eqs (6–8) describe the dynamic of free IL2Rα, IL2Rβ and γc on the cell membrane. The first and second terms of Eqs (6–8) correspond to the constitutive expression of new receptors and internalization by cells. Third and fourth terms in (eq 6) correspond to the interaction between IL2Rα and the complex IL2-IL2Rβ, and fifth and sixth terms to the interaction between IL2Rα and the complex IL2-L2Rβ-γc. Third and fourth terms in (eq 7) correspond to the interaction between IL2Rβ and the complex IL2-IL2Rα. Third and fourth terms in (eq 8) describe the interaction between γc and the complex IL2-L2Rβ, and fifth and sixth terms the interaction between γc and the complex IL2-IL2Rα-L2Rβ.

(Eq 9) describes the dynamics of the number of internalized IL2 molecules per cell. The first term correspond to the internalization of bound IL2 due to the constitutive internalization of IL2Rs. The second term corresponds to ligand induced internalization.

(Eq 10) corresponds to mass conservation of total IL2 in the system. In this equation, *N*_*A*_ is the Avogadro's number, *N*_cells_ and *V* are the total number of cells and the volume of the cell containing media respectively.

### Parameters Treatment in the Model

The system of eqs (1–10) has 16 parameters, which are not specific to the cell type, and describe mostly the binding kinetics of IL2 to IL2 receptors intermediates and the internalization process (referred bellow as the first parameter group). It also has 32 cell-type-dependent parameters, which describe the number IL2R chains per cell and the cell surface area (referred bellow as the second group of parameters).

The first group of parameters includes:

*k*_*αL*_, *k*_*−αL*_, *k*_*βL*_ and *k*_*−βL*_ which are the association and dissociation rates for the interaction between free IL2 and IL2Rα or IL2Rβ (see Table 3). The values for *k*_*βL*_ and *k*_*−βL*_ were fixed following experimental data obtained by Surface Plasmon Resonance (SPR). The values for *k*_*αL*_, and *k*_*−αL*_ could not be set to the values estimated by SPR, since it will prevent the proper fitting of second phase Scartchard plot data in cell lines where IL2Rα is in excess. Therefore the value of *k*_*αL*_ was fixed in the estimated by SPR and the value of *k*_*−αL*_ was slightly corrected from the one obtained by SPR, following fitting of the second phase of several independent Scartchard plots. With this new value the resultant affinity for IL2 binding to IL2Rα (51 nM) is close to the range estimated by Robb [14] (25~45 nM).

*k*_int_ and $k_{\text{int}}^{\text{sig}}$: which are the rates for the constitutive and ligand induced internalization, respectively. The value of *k*_int_ was assumed equal for all IL2R chains, i.e the value estimated for IL2Rβ [29]. The value for $k_{\text{int}}^{\text{sig}}$ is assumed equal for both signalling complexes, IL2-IL2Rβ-γc and IL2-IL2Rα-IL2Rβ-γc, and fixed in the value estimated by Fallon [28].

*k*_*αLβ*_, *k*_*−αLβ*_, *k*_*βLα*_, *k*_*−βLα*_, *k*_*βγLα*_, *k*_*−βγLα*_, *k*_*βLγ*_, *k*_*−βLγ*_, *k*_*αβLγ*_, and *k*_*−αβLγ*_,: which are the kinetic rates of the processes of association and dissociation of intermediate IL2-IL2R complexes on the cell membrane (see Table 3). The values of these parameters are unknown and therefore estimated in the model fitting. It was assumed equal kinetic rates for the interaction of IL2Rα with IL2-IL2Rβ and IL2-IL2Rβ-γc complexes (*k*_*αβLγ*_ = *k*_*βLγ*_; *k*_*−αβLγ*_ = *k*_*−βLγ*_). It was also assumed equal kinetic rates for the interaction of γc with IL2-IL2Rβ and IL2-IL2Rα-IL2Rβ complexes (*k*_*βγLα*_ = *k*_*βLα*_; *k*_*βγLα*_ = *k*_*−βLα*_). These assumptions are based in the fact that extracellular domains of IL2Rα does no interact with γc.

The second group of parameters corresponds to: the equilibrium number of IL2 receptors components per cells in the absence of IL2 ($N_{\alpha 0}^{i}$, $N_{\beta 0}^{i}$,$N_{\gamma 0}^{i}$) and the cell membrane area (*A*^*i*^). These parameters are treated in the model as unknown parameters to be fitted. Although, for the number of IL2Rs, a rough estimation exists from the intercept analysis of some Scarchard plots (see column 3 on Table 3). The value of the cell membrane area (*A*^*i*^) is always referred in relative terms to the one of stimulated blast cells (cell A2).

In model fittings, unknown parameters were constrained within a reasonable, but wide, range of values (see Table 3). The upper limit of association rates (*k*_*αLβ*_, *k*_*βLα*_, *k*_*βLγ*_) was set to the theoretical diffusion limits for the IL2R chains moving in the cell membrane (see S1 File); and their lower limit was taken three orders of magnitude below. The upper limits for the dissociation rates of IL2Rα and IL2Rβ from the intermediates IL2-IL2R complexes (*k*_*−βLα*_, *k*_*−αLβ*_) were set respectively to the dissociation rates of IL2 from IL2Rα and IL2Rβ in solution. The lower limit of these parameters was set in two order of magnitude below. The numbers of IL2R per cell were explored in wider ranges than those originally estimated in the literature (from Scatchard plot intercepts). The relative cell membrane area was explored between 1 and 10.

### Experimental data

In order to calibrate the model it was used the results of IL2 binding assays to different human cell or cell lines. In these assays, the IL2 is added to the cells and after some time (mostly 20 min) the experiment is stopped and the number of IL2 associated to the cells is estimated.

In the case of our system, this type of data contains information regarding the dynamic of IL2-IL2R complexes assembling in the cell membrane, which depends on the density of IL2Rs in the cell surface and the kinetic rates describing the reaction network. This information is related to the apparent affinity corresponding to different phases of Scatchard plot. On the other hand, this data contains information about the IL2 capture from solution, which only depends on the number of IL2Rs and is related with the intercept of Scatchard plot phases with the x axis. This infomation decouples the parameters of the number of IL2Rs with those parameters characterizing the dynamic of IL2-IL2Rs interaction in the cell membrane. Additionally, the assumption of membrane area equal to 1 for A2 cells should decouple the information about kinetic rates and the membrane areas, allowing their independent estimation. Because of the explained above, it was considered that this is the best data for the calibration of the unknown parameters of the model.

The IL2 binding assay corresponding to the used data for the model fitting takes 20 min after IL2 addition to the media (except for B1 and B4 that take 10 and 90 minutes respectively). According to the authors, this time period is sufficient to reach equilibrium, a precondition for a proper Scatchard plot analysis [9,12,30]. The volume of the system is typically 100 μL (200 μL for B2 and B3 cells) and the total number of cells is 10^6^ in all cases. All the studied cells are of human origin. The reported temperature of the assays is 37°C.

### Model Fitting and Parameters Estimation

In order to simulate the typical binding assay, the system of eqs (1–10) is numerically solved with the following initial conditions:

$$\begin{array}{l} N_{\alpha }^{i}\left(0\right)=N_{\alpha 0}^{i},\;N_{\beta }^{i}\left(0\right)=N_{\beta 0}^{i},N_{\gamma }^{i}\left(0\right)=N_{\gamma 0}^{i}\\ N_{\alpha L}^{i}\left(0\right)=N_{\beta L}^{i}\left(0\right)=N_{\alpha \beta L}^{i}\left(0\right)=N_{\beta \gamma L}^{i}\left(0\right)=N_{\alpha \beta \gamma L}^{i}\left(0\right)=N_{\text{int}}^{i}\left(0\right)=0\\ {\left[L\right]}^{i}\left(0\right)={\left[L\right]}_{0}^{i}={\left[L\right]}_{\text{exp}}^{ij} \end{array}$$ (11)

that correspond to the scenario in which the number of IL2Rs at the *i*-cell membrane is initially in equilibrium, when the specified amount of free IL2 (${\left[L\right]}_{\text{exp}}^{ij}$) is added to the culture at *t =* 0. The *j* index refers to the specific point in the *i*-th Scatchard plot.The predicted number of IL2 molecules bound (or associated) to the cell *i* is then calculated as:

$$\text{teo}IL2_{b/cell}^{i}\left(t_{a}\right)=N_{\alpha L}^{i}\left(t_{a}\right)+N_{\beta L}^{i}\left(t_{a}\right)+N_{\alpha \beta L}^{i}\left(t_{a}\right)+N_{\beta \gamma L}^{i}\left(t_{a}\right)+N_{\alpha \beta \gamma L}^{i}\left(t_{a}\right)+N_{int}^{i}\left(t_{a}\right)$$ (12)

where *t*_*α*_ refers to the time consumed by the binding experiment (10 and 90 min for B1 and B4 cells respectively and 20 min in other case).

The least square method was applied to fit the model to the experimental data. In this method the following chi-square function is defined for the simultaneous fitting of available Scatchard plots.

$$\chi^{2}\left(c1,c2\right)=\sum_{i=0}^{N}\sum_{j=0}^{M_{i}}{\left(\frac{\text{teo}IL2_{b/cell}^{ij}\left(c1,c2\right)-\text{exp}IL2_{b/cell}^{ij}}{\sigma^{ij}}\right)}^{2}$$ (13)

In (eq 13), *N* is the total number of experimental data sets, *M*_*i*_ is the total number of points in the *i*th set and *σ*^*ij*^ is the standard deviation of *j*th experimental point in *i*th set. Standard deviation was taken as 10% of the mean experimental value, as reported by Wang [9]. The variables C1 and C2 correspond to the sets of cell type dependent parameters ($N_{\alpha 0}^{i}$, $N_{\beta 0}^{i}$, $N_{\gamma 0}^{i}$ and *A*^*i*^) and the set of kinetic coefficients for the interactions of different complexes in the cell membrane (*k*_*αLβ*_, *k*_*−αLβ*_, *k*_*βLα*_, *k*_*−βLα*_, *k*_*βLγ*_ and *k*_*−βLγ*_) respectively. The total number of C1 group of parameters is 31 (considering the 8 studied cells and that the cell membrane area of A2 cells is fixed to 1). Adding the 6 C2 parameters, the number of degrees of freedom is 37. The total number of observable point in the experimental data is 144.

Local minimums of chi-square function were numerically searched, using FindMinimum routine of version 9.0 of Mathematica, starting from 1000 randomly generated initial sets of the C1 and C2 parameters values into the ranges explained above. The sets of optimal parameters C1m and C2m that minimize the chi-square function and properly explaining the experimental data were initially selected. For this aim, the relative errors between each theoretically predicted Scatchard plot (for each minimum) and the experimental one were computed by the following formula:

$$E_{ij}=\frac{\text{teo}IL2_{b/cell}^{ij}\left(C1,C2\right)-\text{exp}IL2_{b/cell}^{ij}}{\text{teo}IL2_{b/cell}^{ij}}$$ (14)

Those sets of C1m and C2m predicting more that 90% of the *E*_*ij*_ in the range (-0.1, 0.1), were selected (typically a 100–200 out of the 1000 initial local minima).

To further increase the explored space of good fittings of the model, we apply the Monte-Carlo method for the values of C2 parameters around the good solutions (C1m, C2m). This is, we fixed the selected C1 parameters to C1m and randomly vary the value of C2 parameters by pairs ({*k*_*αLβ*_, *k*_*−βLα*_} or {*k*_*βLα*_, *k*_*−βLα*_} or {*k*_*βLγ*_, *k*_*−βLγ*_}). This perturbation was performed 50000 times for each pair of C2 parameters and each optimal C1m and C2m selected above. Those perturbed sets of parameters, which predict a low relative error were added to our set of potential good solutions (typically 4000).

Last, but not least, the set of potential good solutions were filter out with an equilibrium criteria. This is, the kinetic of IL2 binding to the cells reach or get close to an apparent equilibrium in the total number of IL2 bound at the end of experiment. To quantify the proximity to the equilibrium state, the kinetic of IL2 binding to the cells (initial concentration of 10 pM) were simulated and computed the ratio pr = IL2_b/cell_(20min)/IL2_b/cell_(40min). For all the explored solutions, this kinetics is far from the equilibrium state (low value of pr) in cells with low number of IL2Rα because of the low association rate of IL2 to IL2Rβ. Therefore, only the cells with considerable expression of IL2Rα were considered in the equilibrium criteria. We wipe out those fitting solutions were simulations of IL2 binding to these cells predicts a value of pr lower than 0.85.

From the set of good solutions the C1 and C2 parameters corresponding to the lowest value of chi-square function was selected as the best fit. A confidence interval for each individual parameter was estimated as the range covered from its minimum and maximum values among the sets of all good fitting solutions founded.

### Model validation with different data set of IL2 interaction with cells

To evaluate the predictive power of the model we used the data obtained by Cotari and coworkers in [30]. This is a recent data that corresponds to the study of the phosphorylation of the signal transducer and activator of transcription 5 (pSTAT5) as a consequence of IL2 signaling at single cell level. They measured the variations of pSTAT5 level in a pool of activated T stimulated with IL2 at different concentrations. They found that the EC50 value (IL2 concentration causing 50% STAT5 phosphorylation) inversely correlates with the number of IL2Rα.

To explain Cotari’s data, the model was extended to simulate how pSTAT5 level varies with the total number of signaling complexes (the number of IL2-IL2Rβ-γc (*N*_*βγL*_) plus the number of IL2-IL2Rα-IL2Rβ-γc (*N*_*αβγL*_)). To this aim it was assumed a linear relation of pSTAT5 level and the number of signaling complexes. This is based in the result of this same group that the number of IL2 molecules associated to the cells linearly correlates with the level of pSTAT5 [17]. A new equation was added to the system Eqs (1–10) accounting for the variations of pSTAT5 level due to the signaling complexes formation

$$\frac{d{\left(p\text{STAT}5\right)}^{\text{i}}}{\text{dt}}=\kappa \left(N_{\alpha \beta \gamma L}^{i}+N_{\beta \gamma L}^{i}\right)$$ (15)

The variable *pSTAT*5 represents the number of pSTAT5 molecules into the cell due to the interaction with the IL2. The kinetic rate of STAT5 phosphorylation (parameter *κ*) is undetermined but its value is not required to compute the EC50 value.

We simulate the experiment in which the cells remains in equilibrium in IL2 absence and in the initial time the concentration of the ligand is increased to certain value (the same values used in [31]). Therefore, the initial conditions for this simulation are those described in (eq 11). According to [31], the time consumed by this assay is 20 min. In this case, the IL2 concentration is considered constant during the experiment.

To compute the EC50 value we generate sets of *pSTAT*5 for different concentrations of IL2. The EC50 value is determined as the concentration at *pSTAT*5 is the half of the value obtained at the higher concentration by fitting the generated data to a sigmoid function. The number of IL2Rβ and γc were fixed in the values measured in [31]. As in this experiment was performed using activated T cells, the relative cell membrane area was fixed in 1 (which is just our reference cell). The EC50 value was computed for different values of IL2Rα. This procedure was performed for all the set of C2 parameters in the selected good solutions by the criteria explained above.

## Results and Discussion

### Our extended affinity conversion model properly fits the experimental data

**Fig 2:**
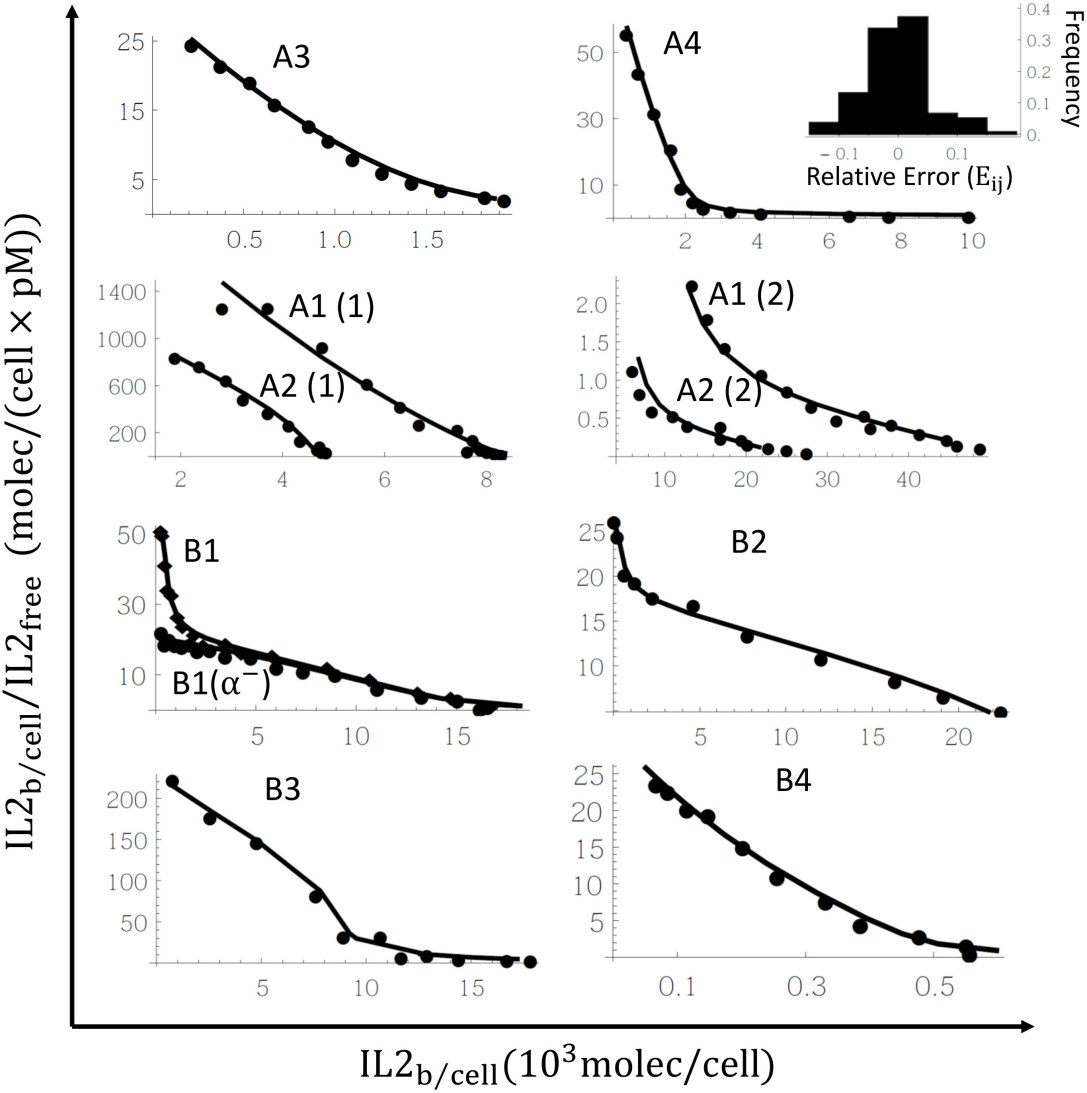
Simultaneous model fitting to the experimental data. Graphs correspond to the Scatchard plots from [9,12,14,32] (dots) and the prediction with the model fitting (solid line). The cell type used in the binding assay is labeled in each graph. The histogram of relative errors (*E*_*ij*_, inserted in the fourth graph) shows a distribution with more than 90% of values in the interval (−0.1, 0.1). The cell dependent and independent parameters (C1 and C2 parameters) in the fitting are resumed in Tables 5 and 6. In panel B1 it is also show the data corresponding to the experiment where the media contains an anti IL2Rα monoclonal antibody that prevents the binding of IL2 to IL2Rα.

We fit our extended affinity conversion model, to the available Scatchard plots data from 8 different cell lines (Table 4). The model was able to fit individually each data set in a wide range of parameters values (data not shown). But more interesting it was also able to fit simultaneously the data for the 8 cell lines. This is assuming that the process of IL2R assembling in the 8 cells is the same, and therefore can be described by the same dynamic model and the same kinetics parameters of IL2 receptor assembling in the membrane. An example of a good simultaneous model fitting is shown in Fig 2. The histogram of relative error frequencies (insert in the figure) shows a distribution where more than 90% of the values lay within the interval (*-0*.*1*, *0*.*1*), confirming the high quality of the fitting.

**Table 4:** Cell lines used in Scatchard Plots.

Cell line	Characterization	Reference
A1	T lymphoblast cell line (HUT102 B2) established from tumor cells derived from the lymph node biopsy.	[12]
A2	Human phytohemagglutinin (PHA)-activated lymphoblasts prepared by culturing isolated PBMC	[12]
A3	Cell line 1C9 obtained by transforming CD25+ B lymphocytes with Epstein-Barr virus	[12]
A4	Activated human T cells prepared by stimulating peripheral blood mononuclear cells with anti T3	[9]
B1	NK cell line (YT) derived from an acute lymphoblastic lymphoma and thymoma patient	[14]
B2	NK cell line (YT) derived from an acute lymphoblastic lymphoma and thymoma patient	[9]
B3	Forskolin induced YT cells	[9]
B4	Acute myelogenous leukemia cell line	[32]

The latter result demonstrates that our model is able to explain the experimental results of IL2 binding to different cells with a diverse distribution of IL2Rs. Furthermore, we proved with several models obtained by simplifying our model and have no success in fitting individual data sets (see S2 File), indicating that this model cannot be simplified without losing the capacity to explain the experimental data. Moreover we demonstrate that the inclusion of γc in the model is necessary for the model explain experimental data, indicating that this chain has a relevant role in the IL2-IL2R interaction.

Furthermore, this result supports the hypothesis of identical kinetic coefficients for all cells. Note that this is not an obvious result, since the movement of IL2R components may vary depending on the composition and structure of the cell membrane, affecting the value of the kinetic coefficients. For instance, it is expected that the differences in the relative presence of IL2Rs components on the lipids rafts also affect the homogeneity in the IL2Rs dynamic between different cells.

### Estimated Values of Cell Dependent Parameters

**Fig 3:**
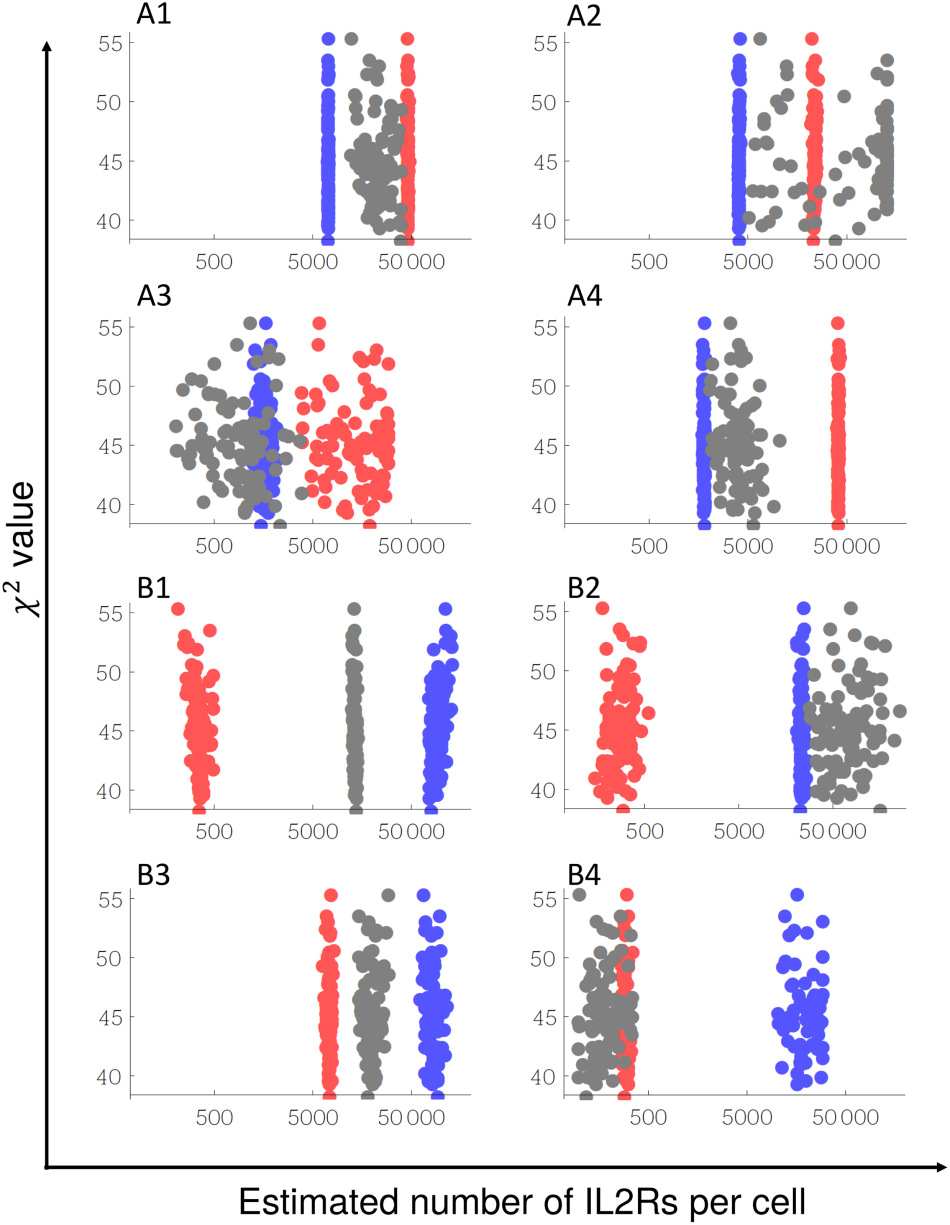
Estimation of the number of IL2Rs per cell. Each point in the graphs correspond to a solution estimated by minimization of chi-square function. The x axis correspond to the estimated number of IL2R subunit (red for IL2Rα, blue for IL2Rβ and gray for γc) and the y axis correspond to the value of chi-square for each solution. The cell type is signaled in the top of each graph.

Fig 3 shows the estimated value of the number of IL2Rs per cell for the 8 studied cells and the corresponding value of chi square function (in the y axis). Each point in the graphs correspond to a good solution selected by the criteria explained in section 3.4. Although the chi-square function has many local minimums (each point correspond to a local minimum), they are relatively close with respect to the value of some of these parameters like the number of IL2Rs in B1 cells (see panel B1 in Fig 3). According to the analysis of the profile likelihood explained in [33] these parameters are structural identifiable despite some of them are not so accurately bounded like the number of γc for A3 and B2 cells ($N_{\gamma }^{A3}0$ and $N_{\gamma }^{B2}0$). The only parameter that appears to be practically no identifiable is the number of γc for A2 cells ($N_{\gamma }^{A2}0$). For this parameter we could only estimate a lower bound.

Table 5 shows the valid ranges of values, predicted out of the model fitting, for the number of IL2Rα ($N_{\alpha 0}^{i}$), IL2Rβ ($N_{\beta 0}^{i}$), γc ($N_{\gamma 0}^{i}$) and the cell surface area (*A*^*i*^) for each cell line after the selection according to the equilibrium criteria explained in the previous section. Table 5 also shows, for comparison, the ranges for the number of low, intermediate and high affinity IL2 receptors estimated by the authors in the original papers by Scatchard method.

**Table 5:** Comparison of estimated range for the number of IL2R components and cell surface are, with the number of low, intermediate and high affinity IL2R estimated from Scatchard plots intercepts.

Cell	Estimation by Scatchard Method			Estimation by Model Fitting to Experimental Data							
	Ranges			Ranges				Best Fit			
	Low	Intermediate	High	*N*_α0_	*N*_β0_	*N*_γ0_	A	$N_{\alpha 0}^{*}$	$N_{\beta 0}^{*}$	$N_{\gamma 0}^{*}$	A*
A1	47600–79200^α^	-	5450–8850^β^	45945–47261^α^	7248–7375^β^	17436–39405	1.8–3.1	47100	7248	39405	2.8
A2	21700–28700^α^	-	2520–4600^β^	23342–24977^α^	4111–4193^β^	27296–130000	1	23342	4144	39187	1
A3	~8200	-	1040–2160^βγ^	10186–29698	1519–1700^β^	1037–2378^γ^	1.8–2.5	19252	1520	2378	1.9
A4	~11500	-	~1900	41349–42073	1836–1878	3930–6652	3.0–4.0	41690	1846	5788	3.2
B1	-	~13100 ^γ^	~1160	315–381	78429–114909	13395–14387^γ^	1.15–3.0	359	80449	14096	1.15
B2	-	~24000^β^	~170	184–300	22921–26278^β^	33878–162164	1.0–2.6	300	22921	162164	2.6
B3	-	~24000^γ^	~8400	7432–7725	92317–105701	15638–27042^γ^	7.3–10.	7489	94151	18398	7.3
B4	-	587–599	147–152 ^γ^	272–318	16948–30000	120–194^γ^	1.0–3.4	294	28024	120	1.0

Superindex α, β or γ indicate an overlapping between the estimated range for the number of low, intermediate or high affinity IL2Rs by Scatchard method and the range for the estimated number of IL2Rα, IL2Rβ and γc through the model fitting to experimental data.

In most cases, and as expected, the estimated number of high affinity IL2 receptors closely corresponds to the smallest number of model predicted IL2R chain component, either IL2Rα, IL2Rβ or γc. In other words, the IL2R chain, which is in defect at the cell membrane, limits the dynamical formation of the high affinity IL2 receptor. Following the same line of reasoning, the number of estimated intermediate affinity receptors seems to match closely the lowest number between the predicted number of IL2Rβ and γc; and the number of low affinity IL2 receptor is mostly similar to the number of IL2Rα chains when they are in a significant excess. However it must be noted that in some cases there is no clear correspondences between the estimated values of the number of low, intermediate and high affinity IL2 receptors estimated from intercepts on Scatchard plots and the IL2R values predicted by model fittings. For instances in the cell A4.

The predicted distribution of IL2Rs shows a wide variety of cases, regarding IL2R composition, among the 8 studied cell lines. For the cells A1 to A4, the number of IL2Rα is larger than the number of IL2Rβ, but for cells B1 to B4 the number of IL2Rβ is larger. There is also a significant diversity regarding the predicted number of γc. Cells A1, A2 and B2 are predicted to have a clear excess of γc in respect to the number of IL2Rβ. But cells A3, B1, B3 and B4 are predicted to have less γc than IL2Rβ at the cell membrane. In some cells the number of γc is comparable to the number of IL2Rβ (A4 cells).

The estimation of cell membrane areas also shows significant differences among the studied cells. In general, the model predicts a relative cell membrane area greater than 1 for all cells (see Table 5). Thus, it predicts that all cells are somehow larger than the A2 reference cells, which correspond to an activated lymphocyte. This result was indeed expected, since immortalized cell lines are typically larger than normal lymphocytes. It is important to note that cells surface area could modulate IL2 receptor dynamic formation at the cell membrane. For most process the concentration of the intermediate species, more than their absolute values, determine the kinetics of the process. In other words, cells with a similar number of IL2Rα, IL2Rβ and γc, but with different surface area, might be expected to capture and signal differently through the IL2R.

Overall the large variation in the number of IL2R components, even in the number of γc, and the cells surface area suggests a fine control of IL2 binding and signaling dynamics on different cells or cell activation states. An interesting case in our data comes from comparing the IL2R and cell surface area between the cells B2 and B3. Cell B3 is obtained by activation of B2 with forskolin. Therefore according to our fitting, the activation of these cells, leads to an expected increase in the number of IL2Rα and IL2Rβ and the cell surface area, but a curious decrease in the number γc. Whether or not the latter is a general phenomenon will require the study of other experimental cases of cell activation.

### Estimation of kinetic coefficients at the cell membrane

The selected solutions from model fitting show variations of around 2 orders of magnitude in the value of the kinetic coefficients, indicating that they can be considered as practical no identifiable according [33].

**Fig 4:**
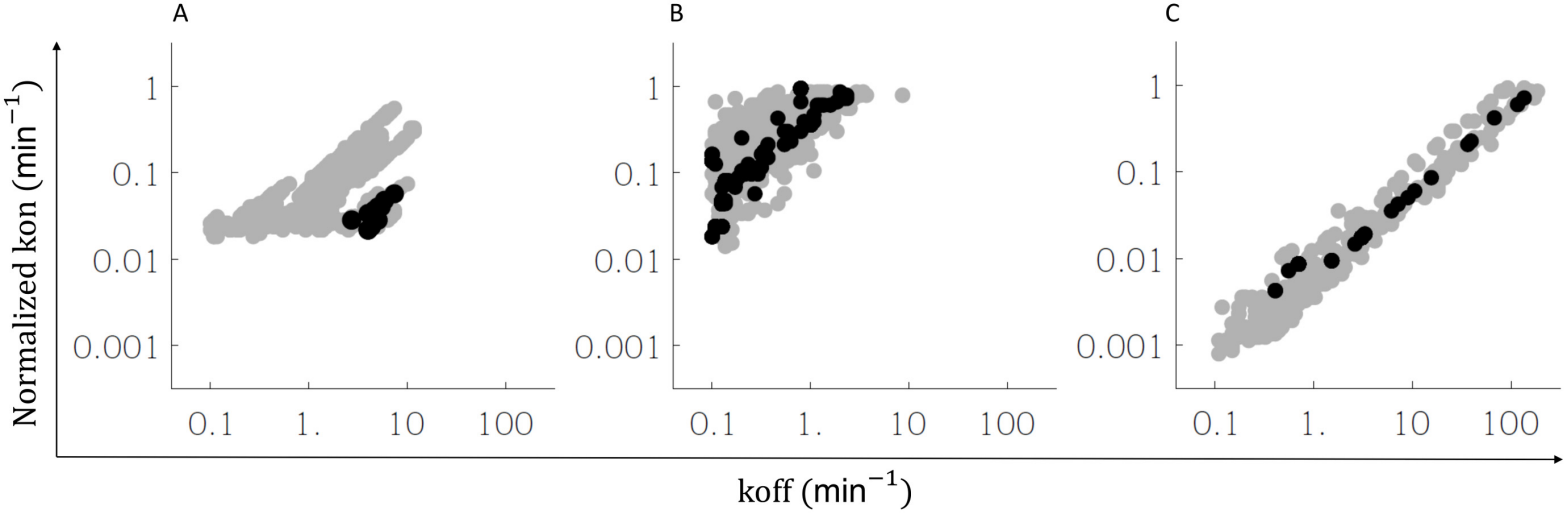
Estimation of kinetic coefficient for the interaction of intermediate IL2R conformations in the cell membrane. Gray zone in each graph corresponds to the pairs of values of {*k*_*αLβ*_, *k*_*−αLβ*_} (panel A), {*k*_*βLα*_, *k*_*−βLα*_} (panel B) and {*k*_*βLγ*_, *k*_*−βLγ*_} (panel C) obtained from a good model fitting selection after application of Monte-Carlo method. The distribution of kinetic coefficients shows a minimal value for the parameter *k*_*αLβ*_ (panel a), a minimal value of the ratio *k*_*βLα*_/*k*_*−βLα*_ (panel B) and a good correlation between *k*_*βLγ*_ and *k*_*−βLγ*_ (panel C). Black zone corresponds to the parameters obtained by applying the equilibrium criteria filter.

The estimated values for the kinetic coefficients for IL2R formation at the cell membrane are shown in Fig 4 and Table 6. Fig 4 has three panels showing the space of good solutions for selected pairs of kinetic coefficients ({*k*_*αLβ*_, *k*_*−βLα*_} or {*k*_*βLα*_, *k*_*−βLα*_} or {*k*_*βLγ*_, *k*_*−βLγ*_}). Each dark point in the graph corresponds to a good fitting solution, including the equilibrium criteria. Each gray point corresponds to a good fitting solution but without applying the last equilibrium filter. As can be seen for all kinetic parameter (*kon* and *koff*) a large range of good fitting values is obtained. The *koff* values span through the whole range in the allowed searched space for the fittings procedure. The *kon* values start from some minimum, which is larger than the lowest value in searched interval, but goes up to the highest allowed value in the searched interval. Interestingly the good fittings, in equilibrium, lay around straight line in the graph. This indicates that the fitting tries to fix the ratio *kon*/*koff*, which by definition correspond to the affinity of the process.

**Table 6:** Comparison of the estimated kinetic coefficient in the cell membrane with the filtered solution by equilibrium criteria.

parameter	Estimated range by goodness of fit criteria	Estimated range by proximity to equilibrium criteria	Best fit
*k*_α*L*β_	2.9 × 10^−4^ − 9.7 × 10^−3^s^−1^	3.4 × 10^−4^ − 9.4 × 10^−4^s^−1^	9.4 × 10^−4^s^−1^
*k*_−α*L*β_	1.7 × 10^−3^ − 0.21s^−1^	3.6 × 10^−2^ − 1.2 × 10^−1^s^−1^	1.2 × 10^−1^s^−1^
$K_{\alpha L\beta }=\frac{k_{\alpha L\beta }}{k_{-\alpha L\beta }}$	4.8 × 10^−3^ − 2.5 × 10^−1^	5.4 × 10^−3^ − 1.1 × 10^−2^	7.8 × 10^−3^
*k*_β*Lα*_(*k*_*βγLα*_)	2.4 × 10^−4^ − 1.5 × 10^−2^s^−1^	2.3 × 10^−3^ − 1.5 × 10^−2^s^−1^	3.5 × 10^−3^s^−1^
*k*_−β*Lα*_(*k*_*−βγLα*_)	1.7 × 10^−3^ − 1.4 × 10^−1^s^−1^	1.8 × 10^−3^ − 2.7 × 10^−2^s^−1^	7.2 × 10^−3^s^−1^
$K_{\beta L\alpha }=\frac{k_{\beta L\alpha }}{k_{-\beta L\alpha }}$	8.5 × 10^−2^ − 8.2	3.0 × 10^−1^ − 2.4	4.9 × 10^−1^
*k*_β*Lγ*_(*k*_*αβLγ*_)	1.3 × 10^−5^ − 1.5 × 10^−2^s^−1^	7.0 × 10^−5^ − 1.2 × 10^−2^s^−1^	1.0 × 10^−3^s^−1^
*k*_−β*Lγ*_(*k*_*−αβLγ*_)	1.9 × 10^−3^ − 2.9s^−1^	6.9 × 10^−3^ − 2.2s^−1^	5.3 × 10^−2^s^−1^
$K_{\beta L\gamma }=\frac{k_{\beta L\gamma }}{k_{-\beta L\gamma }}$	3.2 × 10^−3^ − 2.3 × 10^−2^	5.0 × 10^−3^ − 1.2 × 10^−2^	5.8× 10^−3^

Table 6 shows the values for kinetic coefficients and affinities, estimated by our fittings. We provide the value for the best fit and the range of possible values obtained. Estimated affinities tend to be relatively bounded both in equilibrium and in out of equilibrium good fittings, but smaller confidence intervals are obtained with the equilibrium solutions.

Overall our results show that affinities rather than kinetic coefficients seems to be constrained in the study process. This is an expected result since Scatchard plots are obtained from a typical experiment on equilibrium conditions. It must be expected that the experimental data is less informative regarding the kinetics to reach such equilibrium. In order to get an estimation of the kinetic coefficients a different types of experiment must be designed. For instance our simulations with the model suggests that performing the same binding assay experiment but for two or three different time points (10, 15 and 20 minutes), will provide separate Scatchards plots, whose simultaneous fitting would significantly improve the estimations of individuals *kon* and *koff*. Obtaining an independent measurement of the number of IL2R components in the cell (for instance by flow cytometry based techniques), could also improve the fittings precision. In particular it could help to reduce the range of possible values for the affinities, which so far is constrained inside one order of magnitude.

### Impact of IL2R Internalization in the Model Fitting

In the model it has been taken into account the process of IL2R internalization, both constitutive and induced by the ligand. However previous mathematical models [17,21] have mostly neglected this phenomena. In order to address the impact of this process in our fittings, we perform them again but setting to zero the values of parameters *k*_int_ and $k_{\text{int}}^{\text{sig}}$. The new results are compared with those described above.

**Fig 5:**
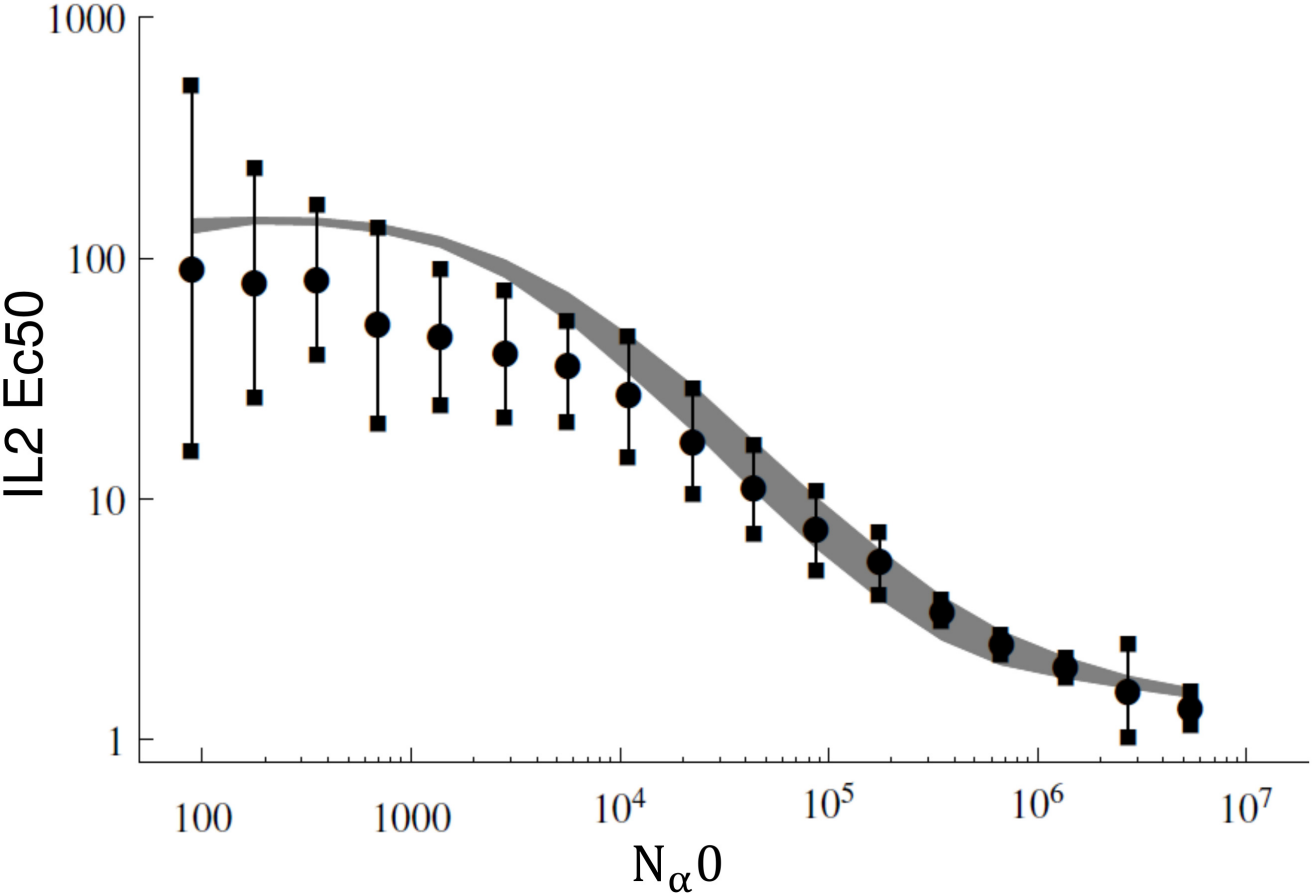
Comparison between experimental measurement and calibrated model prediction of Ec50 dependency on the number of IL2Rα. Dots correspond to the experimental measurements taken from [31]. All the C2 set of parameters obtained by applying the equilibrium criteria filter were evaluated in this simulations and the values of IL2Rβ (*N*_*β*0_) and γc (*N*_*γ*0_) were fixed in 10^3^ per cell and normalized membrane area in 1. The theoretical prediction using the all set of C2 parameters covers the gray zone.

Interestingly, we obtain no differences on the fittings predictions for IL2R binding kinetics, affinities at the cell membrane and surface areas. But a slight overestimation of the number of all IL2R components for each cell was observed (see Fig 5). For instance the predicted ranges for the number of IL2Rα, IL2Rβ and γc in A1 cells turn out to be (50923–55000), (8021–8363) and (15000–50000) respectively. They conserve the same proportions as our initial fitting, but increase slightly by 1.1~1.4 folds. This relative independence of the fitting with the IL2R internalizations was rather unexpected to us, since the characteristic time of these process (15 min) is pretty close to the experimental time in the binding assays (20 min). We believe that the reduced impact of this process derives from two facts: 1) that internalized IL2 still counts as bound IL2 in the experiment (radiation of this label IL2 remains in the cell), and 2) that the actual number of IL2R in the membrane is significantly reduced during the experiment. These two facts combine to reduce the impact of these phenomena to a simple shift in the estimation of the number of IL2R.

On the other hand, internalized complex account in the measurement as a receptor that does not dissociate from the ligand, which is equivalent to a higher affinity complex. This phenomena may affect the estimation of kinetic coefficients. In the case of IL2, only intermediate and high affinity receptors exhibit a high internalization rate. The internalization process does not significantly influence in the apparent affinity due to it is already high.

Additionally, we simulate the possible effect of IL2Rα-mediated recycling in the parameters estimation. We compare the model considering or not the recycling process in the estimated number of IL2Rs per cell and the kinetic coefficients (S3 File), obtaining no significant differences in the values of the estimated parameters. The results obtained in this section prove the robustness of the model to explain the studied experimental data. Nevertheless, the fact that internalization process seem to be irrelevant for model fitting to IL2 binding assay results, it does not mean that they also irrelevant in longer time-scale processes. In fact, Fallon [28] predicts that the consume and recycling process determines the proliferation capacity of T cells in *in vitro* experiments during 72 hours.

### Validation of our Fitted Model with an Independent Data Set

We evaluate whether or not our model of IL2-IL2R binding, once calibrated, can be used to study a completely different experimental context. To this aim we use our model to explain the data obtained by Cotari in [31] as explained in section 3.5.

We obtained that our model qualitatively and quantitatively agree with Cotari’s data. This result is shown in Fig 5, where the gray area is covered by the model predictions using different set of C2 parameters from selected good solutions. Note that this area clearly overlaps with the experimental data (dot points and error bars), especially in the range corresponding to high number of IL2Rα.

Overall this result highlights the validity of our calibrated model. Interestingly, in [31] several mathematical models were proposed to explain this and other data regarding IL7R. They were quite complex models, including preformation of different dimers of IL2R intermediates (IL2Rα-IL2Rβ and IL2Rβ-γc and even IL2Rα-IL2Rβ-γc). However we show here that to explain IL2-IL2R binding in this data an affinity conversion model is good enough. Moreover our independently estimated values of kinetic parameter for IL2R assembly at cells membranes seem to behave properly in other set of cells. This is more relevant taking into account that the Cotari’s experiments were performed with murine lymphocytes and murine IL2.

### Evaluation of the use of different pathways of IL2-IL2R complex formation by different cells

In this section the relevance of the three different pathways of IL2R assembling included in the ACM (Fig 1) is explored for cells with different IL2R configurations. We simulate, with the calibrated model, the kinetics of IL2 binding to cells where particular pathways was individually blocked at the cell membrane. This is, during the solution of the system eqs (1–10), the value of *kon* for the second step of each pathway is set to zero: i.e *k*_*αLβ*_ = 0 for the pathway i, *k*_*βLα*_ = 0 for the pathway ii; and *k*_*βLγ*_ = 0 for the pathway iii. Note that in these simulations, the capture of IL2 from solution is not affected in principle. The contribution of each particular pathway to the IL2 binding kinetics is evaluated, by the ability of the remaining ones to compensate its absences.

**Fig 6:**
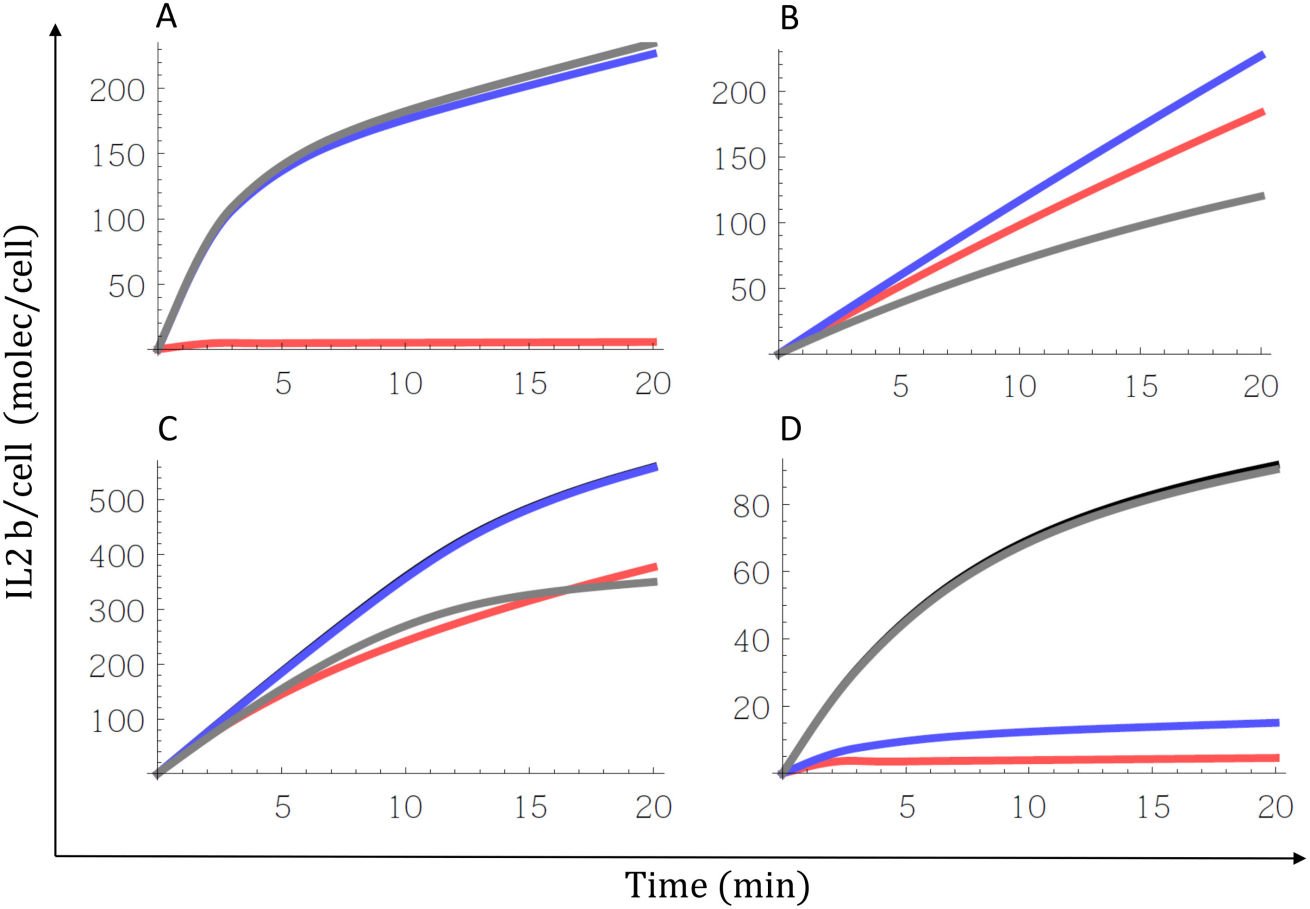
Relevance of pathways for IL2-IL2R assembling. Graphs show the comparison of the kinetics of IL2 binding to different cells (black line) with the kinetics in which a specified pathway is blocked (pathway i (red line) by making *k*_*αLβ*_ = 0, pathway ii (blue line) by making *k*_*βLα*_ = 0 and pathway iii (black line) by making *k*_*βLγ*_ = 0). Panel A, B, C and D correspond to the A1, B2, B1 and B4 cells respectively.

Fig 6 shows the result of these simulations, for four representative cell types. Panel A, correspond to a cell with significant number of IL2Rα compared with IL2Rβ (IL2Rα > 0.1 IL2Rβ), like A1-A4 and B3 cells. In this cell blocking the pathway i (binding free IL2 first to IL2Rα, then to IL2Rβ and then to γc) but not the others two affect the predicted IL2 binding kinetics. This indicates the relevance of pathway i for this cell type. Panel B, corresponds to a cell with IL2Rβ >> IL2Rα and γc > IL2Rβ (B2 cells). In this cell blocking the pathway iii (binding free IL2 first to IL2Rβ, then to γc and then to IL2Rα), but not the others two affect the predicted IL2 binding kinetics. This indicates the particular relevance of pathway iii in this second cell type IL2-IL2R assembling. Panel C correspond to a cell with IL2Rβ and γc >> IL2Rα but with γc < IL2Rβ (B1 cells). In this cell, the kinetic of IL2 binding to the cells is partially affected when both pathway i or iii are blocked, indicating a combination between these two pathways in the IL2-IL2R assembling. Panel D correspond to a cell with IL2Rβ >> IL2Rα but with γc << IL2Rβ (B4 cells). In this cell, the kinetic of IL2 binding to the cells is partially affected when both pathway i or ii are blocked, indicating a combination between these two pathways in the IL2-IL2R assembling.

As IL2Rα is the higher affinity IL2R component and it is highly expressed in many cell types, it is logical that the pathway i is the most accepted pathway for the IL2-IL2R assembling. Here we obtained that the capture of IL2 by IL2Rβ can be relevant for cells with higher expression of IL2Rβ than IL2Rα. In this case the number of γc determines not only the use of ii and iii but also the use of i pathway (corresponding to the capture of free IL2 by IL2Rα), indicating that γc controls the assembling mechanism despite it does not participate in the free IL2 capturing.

### Evaluation of the impact of γc numbers in IL2 capturing and signaling

The quantitative influence of IL2Rα and IL2Rβ in cell signaling capacity and sensitivity have been previously studied [17,31]. They observed that the increase on the number of IL2Rα implies an increase in the cell signaling capacity and an increase in the cell sensitivity (a decrease in the EC50 value for IL2 stimulation). On the other hand, the increase on the number of IL2Rβ also leads to an increase in the cell signaling capacity, but just with a slight variation in the EC50 value. Similar results are obtained with our model (data not shown). However, little is known about the quantitative impact of γc on the capacity of cells to capture IL2 and signal through IL2R. Therefore in this section we use our calibrated model to evaluate such contribution.

**Fig 7:**
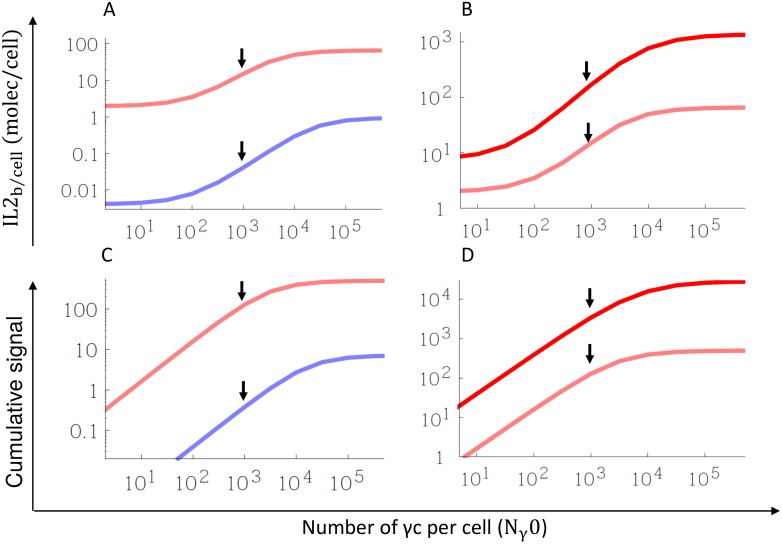
Dependency of IL2 capture and IL2R signaling on the number of γc (*N*_*γ*_0). Panel A and B show the comparison of IL2 bound and cumulative signaling 20 min after IL2 stimulation, for cells with higher expression of IL2Rα (*N*_*α*0_ = 10000; *N*_*β*0_ = 1000, red line) or higher expression of IL2Rβ (*N*_*α*0_ = 0.; *N*_*β0*_ = 1000, blue line). Panel C and D show the comparison of IL2 binding and signaling, at 8 hours after IL2 stimulation for these cells. The concentration of IL2 is considered constant at 1 pM. Arrows indicate the number of IL2Rβ (*N*_*γ*0_ = *N*_*β*0_).

We simulate the IL2 binding to cells with different expression of γc (*N*_*γ*_0), but constant values of IL2Rα (*N*_*α*_0) and IL2Rβ (*N*_*β*_0), at constant concentration of 1 pM. Fig 7 shows the dependency with γc, of the number of IL2 bound per cell (panel a) and the cumulative signaling IL2Rs (calculated through (eq 15) by making *κ* = 1; panel C) after 20 min of IL2 stimulation.

We predict a significant increase in both the capturing and signaling capacity of the cell associated to the increase of the number of γc. In the absence of γc, the IL2 capture is due by the IL2Rα and IL2Rβ receptor, but no signaling is possible. In the excess of γc the IL2 capture and signaling reach a maximal value related to the number of IL2Rα and IL2Rβ in the membrane.

The simulations predict a different impact of the number of γc in cells with high expression of either IL2Rα or IL2Rβ in the IL2 binding but similar behavior in the signaling capacity. In cells with IL2Rα >> IL2Rβ the amount of captured IL2 is affected in around an order of magnitude (Fig 7 panel A, red line) with the increase of γc. This variation is around than the one reported between the apparent affinity of high-(trimeric IL2-IL2Rα-IL2Rβ-γc) and pseudo-high-affinity (dimeric L2-IL2Rβ-γc) IL2Rs (one order of magnitude). In cells with IL2Rα << IL2Rβ the amount of captured IL2 can increase in around two orders of magnitudes with the increase of γc (Fig 7 panel A, blue line). This increment is consistent with the observed variations in apparent affinity between intermediate affinity IL2R and IL2Rβ. In both types of cells, especially in cells with higher number of IL2Rβ, the dependency of the IL2 signaling shows similar sensibility to γc variations. Interestingly, this sensitivity extends for values of γc around 10–100 times the number of IL2Rβ.

In order to simulate a scenario more like *in vivo* scenario we explore the impact of γc variations in the interaction of cells with IL2 for longer periods of time. For that we extended the previous simulation to 480 min (8 hours) corresponding with the doubling time of T cells after antigenic stimulation [34].

The IL2 binding and cumulative signaling at 8 hours is greater than obtained at 20 min but with the similar dependency on the number of γc. This result is shown for both types of cells in Fig 7 panels B and D. Interestingly, this difference of IL2 binding and cumulative signaling between 20 min and 8 hours becomes greater with the number of γc, indicating a higher impact of this component of IL2R for the interaction of cells with IL2 in this scenario.

The biological relevance of γc in IL2/IL2R interaction is also given by the variations in the γc expression that may occur in the cells in different scenarios. This aspect has been poorly studied. However, in the lymphocytes population around 10^3^ copies of γc per cell where measured by Cotari [31]. Our results predict that this number can reach to 10^5^, indicating that in T cells this number may variates in around two orders of magnitude during activation. On the other hand, the results of Voss [35] indicate that human NK cells have around 10^3^ copies of γc per cell, and we obtain that this number in an NK cells lines (YT) can reach to 10^5^, which also suggest variations in around two orders of magnitude.

Feinerman and coworkers [17] measured two orders of magnitude in the saturating level of pSTAT5 response in a pool of activated blast T cells. They associate this result with the variations in the IL2Rα and IL2Rβ expression. According to the model predictions, the variation of γc expression in T and NK cells may promotes similar variations in pSTAT5 level than the variations of IL2Rα and IL2Rβ, indicating that γc (in addition to IL2Rα and IL2Rβ) is an active controller of IL2R signaling.

## Concluding Remarks

We developed a model for the assembling of high affinity IL2-IL2R complex aiming to understand how IL2 interacts with the cells. The model includes the minimal aspects of the known biology of this system, and it was demonstrated that this is the minimal model that explains the available experimental data of IL2 binding to the cells. The model was calibrated by fitting to experimental data, which allow to determine the number of IL2Rs, the membrane area and the kinetic coefficients describing the reactions of intermediate IL2-IL2R complexes. Our main prediction is that the interaction of IL2 with the cells depends on the three chains of IL2R.

It should be noted the role of γc in this interaction since mostly of the publications around the topic refers to IL2Rα and IL2Rβ as the principal mediators of the IL2 biological functions [2,36] and the references therein. Other direct consequence of the relevance of γc in the IL2-IL2R interaction is that the use of γc by other cytokine receptors affects the interaction of cells with IL2, even in cells with excess of γc. This idea has been shared by other authors [31,37,38].

The calibrated model can be used for modeling other phenomena regarding the IL2 interaction with the cells. An interesting example is the IL2 derived muteins that have being designed to increase or decrease the affinity for some IL2R component ([39,40] for IL2Rα, [27,41] for IL2Rβ, [42] for γc and [43] for combinations). The model can extended to simulate the competition of these molecules with the wild type variant for the binding to IL2Rs in different cells, which is very important for the antagonist molecule with decreased affinity for the γc subunit [42].

The main limitations of the model are given by the fact that the IL2-IL2R interaction can be more complex. On one hand several experimental findings suggest that the IL2Rs can be associated forming some dimers before ligand binding ([24] between IL2Rα and IL2Rβ; and [25] between IL2Rβ and γc). On the other hand, some experimental findings suggest the location of IL2R subunits inside membrane microdomains such as lipid rafts. In different cell types some authors have found that these microdomains are enriched of IL2Rα [25,44], IL2Rβ [45], and even the three IL2R subunits [46,47]. The inclusion of these processes leads to a more complex model with higher number of unknown parameters. In example the effective membrane area for lateral diffusion of IL2-IL2R complexes cannot be associated to the cell membrane area. Presumably, the resulting model, as well as our, can properly fit the experimental data, which means that the available data is not sufficient to distinguish between our model and this more complex model. Therefore, other additional data should be needed for the model calibration

## Supporting Information

S1 File Kon limits due to the diffusion rate. (DOCX)

S2 File Effect of simplifications in the model fitting. (DOCX)

S3 File Effect of IL2Rα-mediated recycling in the estimated parameters by model fitting. (DOCX)

S1 Fig Dependence of initial slope of Scatchard pot with the number of low affinity IL2Rs. (TIF)

S2 Fig Effect of the simplification of the model neglecting reactions corresponding to capture from solution in the capacity to explain the experimental data. (TIF)

S3 Fig Simplification of the model deleting reactions in the cell membrane. (TIF)

S4 Fig Best fit of model considering an excess of γc to B1 cells data. (TIF)

S5 Fig Comparison of the estimated parameters by fitting the model considering or not the internalization process. (TIF)

S6 Fig Comparison of the estimated parameters by fitting the model considering or not the IL2Rα mediated recycling. (TIF)
