# Supplementary material for: Quantitative Contribution of IL2Rγ to the Dynamic Formation of IL2-IL2R Complexes
Source: PLoS One. 2016 May 19;11(5):e0155684. doi: 10.1371/journal.pone.0155684 (PMC4873224; doi:10.1371/journal.pone.0155684)
Supplement: S1 File — (DOCX) [file pone.0155684.s001.docx]

# Kon limits due to the diffusion rate

The rate of chemical elementary reaction cannot surpass the diffusional barrier of the interacting species. The procedure followed to estimate this limit for the *kon* values of membrane-occurring processes is presented here.

The diffusion-controlled collision frequency, i.e. the number of collisions per *cm^2^* per second (${N_{col}}/\left( {cm}^{2}s \right)$), between two molecules A and B in a 2-D surface is given by the following equation [[1](#_ENREF_1)]

$\frac{N_{\text{col}}}{cm^{2}s}=2 \pi\frac{N_{a}N_{b}}{s^{2}}[\frac{D_{a}}{\log\left( \frac{1}{r_{ab}}\left( \frac{\pi N_{b}}{s} \right)^{-\frac{1}{2}} \right)}+\frac{D_{b}}{\log(\frac{1}{r_{ab}}\left( \frac{\pi N_{a}}{s} \right)^{-\frac{1}{2}})}]$ (1.1)

where *N_a_* and *N_b_* stand for the number of A and B, respectively. *D_a_* and *D_b_* represent the lateral diffusion coefficients for A and B, expressed in cm^2^/s. Finally, *r_ab_* is the sum of the radio of the interacting molecules, and *S* is the area of the 2-D surface.

The rate of the reaction between A and B to form AB, can be expressed in terms of the number of AB molecules produced per *cm^2^* per second (${\text{N}_{\text{ab}}}/\left( \text{cm}^{\text{2}}\text{s} \right)$). The latter variable is expressed as a function of *N_a_*, *N_b_* and the rate constant *kon_ab_*:

$\frac{N_{ab}}{cm^{2}s}=kon_{ab}\frac{N_{a}N_{b}}{s^{2}}$ (1.2)

The maximum rate value is reached when every collision yields an AB molecule. Therefore, by equating (1.1) and (1.2), the diffusional limit of the rate constant (*kon_ab(lim)_*) can be expressed by the following equation:

$kon_{ab(\text{lim})}=2 \pi[\frac{D_{a}}{\log\left( \frac{1}{r_{ab}}\left( \frac{\pi N_{b}}{s} \right)^{-\frac{1}{2}} \right)}+\frac{D_{b}}{\log(\frac{1}{r_{ab}}\left( \frac{\pi N_{a}}{s} \right)^{-\frac{1}{2}})}]$ (1.3)

Note that (*kon_ab(lim)_*) weakly depends on the values of *N*a and *N*b and linearly depends on *D*a and *D*b values. To estimate the value of *kon_ab(lim)_* for the bimolecular events between IL2R species on the cell membrane it was assumed that:

$\text{D}_{\text{a}}=\text{D}_{\text{b}}=\text{2}\times\text{10}^{-\text{10}}{\text{cm}^{\text{2}}}/\text{s}$The lateral diffusion coefficient of α chain was determined by *Fluorescence Recovery After Photobleaching* (FRAP) [[2](#_ENREF_2)]. It was assumed that the lateral diffusion coefficients for the other species are the same. More recently, the lateral diffusion coefficients for IL2R chains cells have been determined in Kit-225 cells [[3](#_ENREF_3)], resulting that they are an order of magnitude greater. We demonstrate that our results are fulfilled even considering the Edidin lateral diffusion limit.

$\text{r}_{\text{ab}}=\text{5.3nm}=\text{5.3}\times\text{10}^{-\text{7}}\text{cm}$ This value was calculated by using a formula that relates the spherical radius of a globular protein with its molecular weight, i.e. [[4](#_ENREF_4)]. Molecular weights of IL2Rα, IL2Rβ and γc are 55, 75 and 65 *kDa*. The presence of the ligand increases the total molecular weight in 15 kDa, changing the *r* value around 0.1 *nm*. In the case of dimers the *r* value can be increased in ~ 1 *nm,* but this does not translate into a significant change in *kon_ab(lim)_* value.

$\text{N}_{\text{a}}=\text{30000 , Nb}=\text{5000}$ These are the approximated values of the number of IL2Rα and IL2Rβ respectively in PHABL cells. Other values of IL2Rα, IL2Rβ or γc can be used in (1.3) but no significant modifications would be obtained in the value of *kon_ab(lim)_* .

$\text{s}=\text{2.5}\times\text{10}^{-\text{6}}\text{cm}^{\text{2}}$ This value was calculated considering the PHA-BL cell as a sphere with 9 μm of diameter [[5](#_ENREF_5)].

# References

1. Hardt S (1979) Rates of diffusion controlled reactions in one, two and three dimensions. Biophys Chem 10: 239-243.

2. Edidin M, Aszalos A, Damjanovich S, Waldmann TA (1988) Lateral diffusion measurements give evidence for association of the Tac peptide of the IL-2 receptor with the T27 peptide in the plasma membrane of HUT-102-B2 T cells. J Immunol 141: 1206-1210.

3. Pillet AH, Lavergne V, Pasquier V, Gesbert F, Theze J, et al. (2010) IL-2 induces conformational changes in its preassembled receptor core, which then migrates in lipid raft and binds to the cytoskeleton meshwork. J Mol Biol 403: 671-692.

4. Venturoli D, Rippe B (2005) Ficoll and dextran vs. globular proteins as probes for testing glomerular permselectivity: effects of molecular size, shape, charge, and deformability. Am J Physiol Renal Physiol 288: F605-613.

5. Decoursey TE, Chandy KG, Gupta S, Cahalan MD (1987) Mitogen Induction of Ion Channels in Murine T Lymphocytes J Gen Physiol 89: 405-420.
