# Supplementary material for: Quantitative Contribution of IL2Rγ to the Dynamic Formation of IL2-IL2R Complexes
Source: PLoS One. 2016 May 19;11(5):e0155684. doi: 10.1371/journal.pone.0155684 (PMC4873224; doi:10.1371/journal.pone.0155684)
Supplement: S2 File — (DOCX) [file pone.0155684.s002.docx]

# Effect of simplifications in the model fitting

In this section it is presented the results of the evaluation of others models in the literature and models obtained by reducing the model with logical simplification with respect to the capacity to explain the available experimental data. It is demonstrated that any simplification of our model leads to the loss of the model capacity to individually or simultaneously explain all the experimental data.

## The model of Goldstein

Goldstein and coworkers [[1](#_ENREF_1)] proposed a model of IL2R assembling in which the γc is not considered and the IL2Rβ is assumed to interact with the IL2 as the intermediate affinity IL2R. They assume that IL2Rα and IL2Rβ should be performed before the ligand binding. In our model this assumption is equivalent to a fast association between IL2-IL2Rα complex and IL2Rβ. Goldstein demonstrate that as a consequence of this assumption, the slope of high affinity phase of Scatchard plot is constant and independent on the number of IL2Rs.

Some experimental data analyzed by Goldstein show low variations in the initial slope of Scatchard plot as shown in S1 Fig black points, support the predicted consequence. However, the data analyzed by Goldstein correspond to cells with large number of IL2Rα. Therefore it was decided to search for more experimental data corresponding also to cells with low number of IL2Rα. It was found a larger variations in the initial slope of Scatchard plot corresponding to other cells. As shown in S1 Fig gray points, the cells with lower number of IL2Rα, including those analyzed by us, do not comply with the Goldstein prediction.

## Simplifications of the reaction network of the model

In this section the possible model simplifications based on the neglecting of reaction from 1-7 net was explored. To accomplish the simplification, the value of kon and koff corresponding to the reaction were set to zero. It was found that the simplified models are unable to individually fit some experimental data.

### The model neglecting the reactions corresponding to the ligand capture from solution

The model was simplified by neglectin the reactions corresponding to the capture of IL2 by IL2Rα (reaction R1) or IL2Rβ (reaction R2), obtaining that these model cannot explain the data corresponding to the first phase of Scatchard plot of A1 cells and the Scatchard plot of B1 cells respectively. This result is shown in S2 Fig, which shows the best fit of the model neglecting reaction R1 to the data corresponding to A1 cells in panel A and the best fit of the model neglecting the reaction R2 to de data corresponding to B1 cells in panel B.

### The model neglecting reactions in the cell membrane

We simplified the model by neglecting the interaction between the complex IL2-IL2Rα and IL2Rβ (reaction R3) or the complex IL2-IL2Rβ and IL2Rα (reactions R5 and R7). In this cases the values of $k_{\alpha L\beta}$ and $k_{-\alpha L\beta}$ or $k_{\beta L\alpha}(k_{\beta\gamma L\alpha})$ and$k_{\beta L\alpha}\left( k_{-\beta\gamma L\alpha} \right)$ were set to zero. In both cases the simplification reduces the number of unknown kinetic parameters to 4.

It was obtained that the model neglecting the reaction R3 cannot explain the data corresponding to the cells with high number of IL2Rα. An example is presented in S3 Fig panel A, which shows the best obtained fit to the data corresponding to A2 cells. On the other hand, we obtain that the model neglecting reactions R5 and R7 cannot explain the data corresponding to cells with high number of IL2Rβ. An example is presented in S3 Fig panel B, which shows the best obtained fit to the data corresponding to B1 cells.

The other reactions can be neglected in order to simplify the model are R4 and R6, corresponding to the interaction between IL2-IL2Rβ and γc. The obtained model by this simplification is clearly inconsistent with the experimental data (specially the data corresponding to the A1 cells in the presence or excess of an antibody anti IL2Rα) since IL2Rβ binds the IL2 with $K_{d}\sim{10}^{-7} M$, two orders of magnitude greater than the observed for intermediate affinity phase of Scatchard plot ($K_{d}\sim{10}^{-9} M$).

## The model considering γc chain in excess

Other simplification that has been previously used in the literature is the consideration of γc in excess [[1](#_ENREF_1),[2](#_ENREF_2)]. This consideration implies that the IL2Rβ behaves as the intermediate affinity IL2R. This equivalent receptor should capture the ligand with the same rate ($k_{\beta L}$) than IL2Rβ. This supposition is consistent with the kinetic rates measured for IL2 binding to YT cells (cells expressing high number of IL2Rβ and γc and lower number of α) in [[3](#_ENREF_3)]. The contribution of γc in this model is implicit in the effective value of parameter$k_{-\beta L}$. This simplification reduces the number of kinetic coefficients and cell dependent parameters. On the other hand the model fitting requires the estimation of the parameter$k_{-\beta L}$.

We obtain that this model is consistent with the data corresponding to A1-A4. This is an expected result since the IL2 binding to these cells do not depends on the capture by IL2Rβ. Moreover, this model properly fits the data corresponding to B2 cells. This result is consistent with the fact that for B2 cells we have already estimated an excess of γc over IL2Rβ. On the other hand, this model cannot properly fit the data corresponding to those cells for which we predicted a lower number of γc (B1, B3 and B4 cells) as it is shown in S4 Fig for B1 cells. This result is due to the low kinetic at which IL2 binds to IL2Rβ.

The kinetic rate of IL2 association to co-transfected cells with IL2Rβ and γc genes, measured by Pillet and coworkers [[4](#_ENREF_4)] is$1.8\times{10}^{7}M^{-1}s^{-1}$. This value is two orders of magnitude greater than the measured by SPR technique ($7.9\times{10}^{5}M^{-1}s^{-1}$) [[5](#_ENREF_5)] or the measured by this group using cells only transfected with IL2Rβ gene ($6\times{10}^{5}M^{-1}s^{-1}$) [[4](#_ENREF_4)]. Moreover they measured similar dissociation rate of IL2 from cells transfected with IL2Rβ gene and from cells co-transfected with IL2Rβ and γc genes. This result disagree with the measured by Wang and Smith [[3](#_ENREF_3)] despite being similar experiments (IL2 binding assay in cells expressing intermediate affinity IL2Rs), indicating that γc influences in the IL2 capture but not in IL2 retention. This result cannot be explained by the affinity conversion mechanism and should be understood in the context of performed dimers before ligand binding. Dimers preformation mechanism requires an additional explanation of how IL2R signaling does not occurs once IL2Rβ binds to γc.

# References

1. Goldstein B, Jones D, Kevrekidis IG, Perelson AS (1992) Evidence for p55-p75 heterodimers in the absence of IL-2 from Scatchard plot analysis. Int Immunol 4: 23-32.

2. Feinerman O, Jentsch G, Tkach KE, Coward JW, Hathorn MM, et al. (2010) Single-cell quantification of IL-2 response by effector and regulatory T cells reveals critical plasticity in immune response. Mol Syst Biol 6: 437.

3. Wang HM, Smith KA (1987) The interleukin 2 receptor. Functional consequences of its bimolecular structure. J Exp Med 166: 1055-1069.

4. Pillet AH, Lavergne V, Pasquier V, Gesbert F, Theze J, et al. (2010) IL-2 induces conformational changes in its preassembled receptor core, which then migrates in lipid raft and binds to the cytoskeleton meshwork. J Mol Biol 403: 671-692.

5. Levin AM, Bates DL, Ring AM, Krieg C, Lin JT, et al. (2012) Exploiting a natural conformational switch to engineer an interleukin-2 'superkine'. Nature 484: 529-533.

6. He YW, Adkins B, Fuse RK, Malek TR (1995) Expression and Function of the yc Subunit of the IL-2, IL-4 and IL-7 Receptors. Distinct Interaction of yc in the IL-4 Receptor. J Immunol 154: 1596-1605.

7. Hori T, Uchiyama T, Umadome H, Tamori S, Tsudo M, et al. (1986) Dissociation of Interleukin-2 Mediated Cell Proliferation and Interleukin-2 Receptor Upregulation in Adult T-Cell Leukemia Cells. Leuk Res 10: 1447-I1453.

8. Robb RJ, Greene WC, Rusk CM (1984) Low and high affinity cellular receptors for interleukin 2. Implications for the level of Tac antigen. J Exp Med 160: 1126-1146.

9. Robb RJ, Rusk CM, Yodoi J, Greene WC (1987) Interleukin 2 binding molecule distinct from the Tac protein: analysis of its role in formation of high-affinity receptors. Proc Natl Acad Sci U S A 84: 2002-2006.

10. Robb RJ, Rusk CM (1986) High and low affinity receptors for interleukin 2: implications of pronase, phorbol ester, and cell membrane studies upon the basis for differential ligand affinities. J Immunol 137: 142-149.

11. Schumann RR, Nakarai T, Gruss HJ, Brach MA, von Arnim U, et al. (1996) Transcript synthesis and surface expression of the interleukin-2 receptor (alpha-, beta-, and gamma-chain) by normal and malignant myeloid cells. Blood 87: 2419-2427.

12. Uchiyama T, Hor T, Tsudo M, Wano Y, Umadome H, et al. (1985) Interleukin-2 Receptor (Tac Antigen) Expressed on Adult T Cell Leukemia Cells. J Clin Invest 76: 446-453.

13. Voss SD, Robb RJ, Weil-Hillman G, Hank JA, Sugamura K, et al. (1990) Increased expression of the interleukin 2 (IL-2) receptor beta chain (p70) on CD56+ natural killer cells after in vivo IL-2 therapy: p70 expression does not alone predict the level of intermediate affinity IL-2 binding. J Exp Med 172: 1101-1114.

# Figure Captions

**S1 Fig. Dependence of initial slope of Scatchard pot with the number of low affinity IL2Rs.** Points correspond to the Scatchard plot of different cells. It is plotted the estimated initial slope of Scatchard plots (y axis) and the estimated number of low affinity IL2Rs (x axis), also estimated by Scatchard method. Data correspond to different cell types with the number of IL2Rα in a wide range of values (HUT102B2, PHA-blasts, PBL, MLC blasts, 1C9, KG, Activated T cells, Adult T-cell Leukemia, Acute Myeloid Leukemia) The data was directly taken from the literature [[3](#_ENREF_3),[6-13](#_ENREF_6)] . Black points correspond to the data analyzed in [[1](#_ENREF_1)].

**S2 Fig. Effect of the simplification of the model neglecting reactions corresponding to capture from solution in the capacity to explain the experimental data.** Panel A shows the best fit of the model neglecting the reaction R1 to the data corresponding to A1 cells. In this solution$N_{\alpha}^{A1}0=65000$, $N_{\beta}^{A1}0=159372.$, $N_{\gamma}^{A1}0=7784.$, $A^{A1}=3.3$ and $k_{\alpha L\beta}=0.89 \text{min}^{-1}$, $k_{-\alpha L\beta}=0.15 \text{min}^{-1}$, $k_{\beta L\alpha}=3\times{10}^{-4} \text{min}^{-1}$, $k_{-\beta\alpha L}=1.13 \text{min}^{-1}$, $k_{\beta L\gamma}=0.89 \text{min}^{-1}$, $k_{-\beta L\gamma}=0.67 \text{min}^{-1}$. Panel B shows the best fit of the model neglecting the reaction between R2 to the data corresponding to A1 cells (the first phase of Scatchard plot). In this solution$N_{\alpha}^{A1}0=4337.$, $N_{\beta}^{A1}0=73593.$, $N_{\gamma}^{A1}0=1201.$, $A^{A1}=3.4$ and $k_{\alpha L\beta}=0.09 \text{min}^{-1}$, $k_{-\alpha L\beta}=13.2 \text{min}^{-1}$, $k_{\beta L\alpha}=3.2\times{10}^{-4} \text{min}^{-1}$, $k_{-\beta\alpha L}=0.34 \text{min}^{-1}$, $k_{\beta L\gamma}=0.9 \text{min}^{-1}$, $k_{-\beta L\gamma}=15.1 \text{min}^{-1}$.

**S3 Fig. Simplification of the model deleting reactions in the cell membrane.** Panel A shows the best fit of the model neglecting the reactions R3 to the data corresponding to A1 cells. In this solution$N_{\alpha}^{A1}0=4319$, $N_{\beta}^{A1}0=108930.$, $N_{\gamma}^{A1}0=100.$, $A^{A1}=1$ and $k_{\beta L\alpha}=0.9 \text{min}^{-1}$, $k_{-\beta\alpha L}={10}^{-4} \text{min}^{-1}$, $k_{\beta L\gamma}=3\times{10}^{-3} \text{min}^{-1}$, $k_{-\beta L\gamma}=240. \text{min}^{-1}$. Panel B shows the best fit of the model neglecting the reaction between IL2:IL2Rα and IL2Rβ in the cell membrane to the data corresponding to A2 cells (the first phase of Scatchard plot). In this solution$N_{\alpha}^{B1}0=1698.$,$N_{\beta}^{B1}0=98593$, $N_{\gamma}^{B1}0=14818$, $A^{B1}=1.5$ and $k_{\alpha L\beta}=0.9 \text{min}^{-1}$, $k_{-\alpha L\beta}={10}^{-4} \text{min}^{-1}$, $k_{\beta L\gamma}=0.56 \text{min}^{-1}$, $k_{-\beta L\gamma}=83.6 \text{min}^{-1}$

**S4 Fig. Best fit of model considering an excess of γc to B1 cells data.** Dot points correspond to the experimental data and solid line correspond to the theoretical prediction. In the estimated solution$N_{\alpha}^{B1}0=4159.$, $N_{\alpha}^{B1}0=17684.$, $A^{B1}=1.0$, $k_{-\beta L}=1.0\times{10}^{-4} \text{min}^{-1}$, $k_{\alpha L\beta}=0.7 \text{min}^{-1}.$, $k_{-\alpha L\beta}=1.3\times{10}^{-3} \text{min}^{-1}$, $k_{\beta L\alpha}=0.1 \text{min}^{-1}$,$k_{-\beta L\alpha}=23.4 \text{min}^{-1}$
