# Supplementary material for: Quantitative Contribution of IL2Rγ to the Dynamic Formation of IL2-IL2R Complexes
Source: PLoS One. 2016 May 19;11(5):e0155684. doi: 10.1371/journal.pone.0155684 (PMC4873224; doi:10.1371/journal.pone.0155684)
Supplement: S3 File — (DOCX) [file pone.0155684.s003.docx]

# Effect of IL2Rα-mediated recycling in the estimated parameters by model fitting

## Comparison of the models considering or not the internalization process

In this section it is shown the comparison between estimated membrane kinetic coefficients and the number of IL2 receptors per cells with the models considering or not the internalization process. The model without considering the internalization process is obtained by setting to zero the values of parameters $k_{\mathrm{int}}$ and $k_{\mathrm{int}}^{\text{sig}}$. In this model the number of IL2Rs per cell remain constant in time and equal to the initial value. This simplest model is able to simultaneous fit the data corresponding to the 8 studied cells. We performed the selection of good solutions with the same criteria explained in the section Methods. As it can be seen in S5 Fig the selected good solutions show no significant differences in the estimated values of kinetic coefficients (upper panel) or the number of IL2Rs per cell (lower panel).

## Effect of the IL2Rα mediated recycling in parameters estimation

The relevance of the IL2Rα-mediated recycling of IL2 for the proliferation of T cells were modeled by Fallon and coworkers [[1](#_ENREF_1)]. They concluded that this process may be important for retaining the IL2 in the cell containing media and therefore sustain the stimulation and proliferation. Here we explore the possible effect of IL2Rα-mediated recycling process in the parameter estimation. To this aim we extended the model to include the recycling of IL2Rα and IL2-IL2Rα complexes after ligand induced internalization.

A new equation should be added to the system (1-10) that account for the accumulation of IL2Rα into the cells due to the internalization of high affinity IL2Rs:

$\frac{dN_{\alpha L\mathrm{ind}}^{i}}{dt}=k_{\mathrm{int}}^{\mathrm{sig}} N_{\alpha\beta\gamma L}^{i}-k_{\mathrm{rec}} N_{\alpha L\mathrm{ind}}^{i}$ (s11)

The first term in (s11) correspond to the internalization of high affinity IL2Rs and the second term correspond to the recycling process. The variable $N_{\alpha L\mathrm{ind}}^{i}$ represents the total number of internalized IL2-IL2Rα complexes through the ligand induced internalization process. The internalized complexes through natural internalization is not considered because this is a slower process compared to the ligand induced internalization process. The parameter $k_{\mathrm{rec}}$ is the recycling rate and its value is fixed in $0.15 \text{min}^{-1}$ [[1](#_ENREF_1)]. Equations (1), (6) and (9) are then transformed as:

$\frac{dN_{\alpha L}^{i}}{dt}=k_{\alpha L}N_{\alpha}^{i}\left[ L \right]^{i}-k_{-\alpha L}N_{\alpha L}^{i}-\frac{k_{\alpha L\beta}}{A^{i}}N_{\alpha L}^{i}N_{\beta}^{i}+k_{-\alpha L\beta}N_{\alpha\beta L}^{i}-k_{\mathrm{int}}N_{\alpha L}^{i}+k_{\mathrm{rec}} r_{f} N_{\alpha L\mathrm{ind}}^{i}$ (s1)

$\frac{dN_{\alpha}^{i}}{dt}=k_{\mathrm{int}}N_{\alpha0}^{i}-k_{\mathrm{int}}N_{\alpha}^{i}-k_{\alpha L}N_{\alpha}^{i}\left[ L \right]^{i}+k_{-\alpha L}N_{\alpha L}^{i}-\frac{k_{\beta L\alpha}}{A^{i}}N_{\beta L}^{i}N_{\alpha}^{i}+k_{-\beta L\alpha}N_{\alpha\beta L}^{i}-\frac{k_{\beta\gamma L\alpha}}{A^{i}}N_{\beta\gamma L}^{i}N_{\alpha}^{i}+k_{-\beta\gamma L\alpha}N_{\alpha\beta\gamma L}^{i}+k_{\mathrm{rec}}\left( 1-r_{f} \right)N_{\alpha L\mathrm{ind}}^{i}$ (s6)

$\frac{dN_{\mathrm{int}}^{i}}{dt}=k_{\mathrm{int}}\left( N_{\alpha L}^{i}+N_{\alpha\beta L}^{i}+N_{\beta L}^{i} \right)+k_{\mathrm{int}}^{\mathrm{sig}}\left( N_{\beta\gamma L}^{i}+N_{\alpha\beta\gamma L}^{i} \right)-k_{\mathrm{rec}} r_{f} N_{\alpha L\mathrm{ind}}^{i}$ (s9)

The last term of equation (s1) corresponds to the fraction $r_{f}$ of recycled IL2 mediated by IL2Rα (20 % of the total internalized IL2 as the high affinity complexes, according to the estimation in [[1](#_ENREF_1)]). The last term in equation (s6) corresponds to the recycling of IL2Rα. Note that we considered that all the internalized IL2Rα return to the cell surface, but a fraction $r_{f}$ associated with an IL2 molecule and a fraction ($1-r_{f}$) alone. The last term in equation (s9) corresponds to the variations in the number of internalized IL2 molecules by the IL2Rα mediated recycling.

In order to evaluate the influence of the recycling process in the estimated parameters by model fitting we perform the same procedure explained in section 3.

We obtain that this model properly fit the experimental data and allow us to select the good solutions with the criteria explained in section 3.4. The estimation of kinetic coefficients show no significant differences with respect to the estimated using the model without considering the recycling process. This result is shown in S6 Fig upper panel.

The estimation of the number of IL2Rs show a slight shift in the estimated number of IL2Rs (i.e. the number of IL2Rα in B3 cell) as shown in S6 Fig lower panel.

# References

1. Fallon EM, Liparoto SF, Lee KJ, Ciardelli TL, Lauffenburger DA (2000) Increased endosomal sorting of ligand to recycling enhances potency of an interleukin-2 analog. J Biol Chem 275: 6790-6797.

# Figure Captions

**S5 Fig. Comparison of the estimated parameters by fitting the model considering or not the internalization process.** Upper panel show the estimated number of kinetic coefficients by minimization of chi-square function. The corresponding reaction is labeled in the top of each graph. Gray points correspond to the initial model and black points correspond to the model considering the IL2Rα mediated recycling. Lower panel show the estimated number of IL2Rs for three representative cells (labeled in the top of each graph). The red, blue and gray points correspond to IL2Rα, IL2Rβ and γc respectively. Lighter colors correspond to the fitting with the initial model and darkest correspond to the simplest model.

**S6 Fig. Comparison of the estimated parameters by fitting the model considering or not the IL2Rα mediated recycling.** Upper panel show the estimated number of kinetic coefficients by minimization of chi-square function. The corresponding reaction is labeled in the top of each graph. Gray points correspond to the initial model and black points correspond to the model considering the IL2Rα mediated recycling. Lower panel show the estimated number of IL2Rs for three representative cells (labeled in the top of each graph). The red, blue and gray points correspond to IL2Rα, IL2Rβ and γc respectively. Lighter colors correspond to the fitting with the initial model and darkest correspond to the model considering the recycling
