# Supplementary figures and images for: Quantitative Contribution of IL2Rγ to the Dynamic Formation of IL2-IL2R Complexes
Source: PLoS One. 2016 May 19;11(5):e0155684. doi: 10.1371/journal.pone.0155684 (PMC4873224; doi:10.1371/journal.pone.0155684)

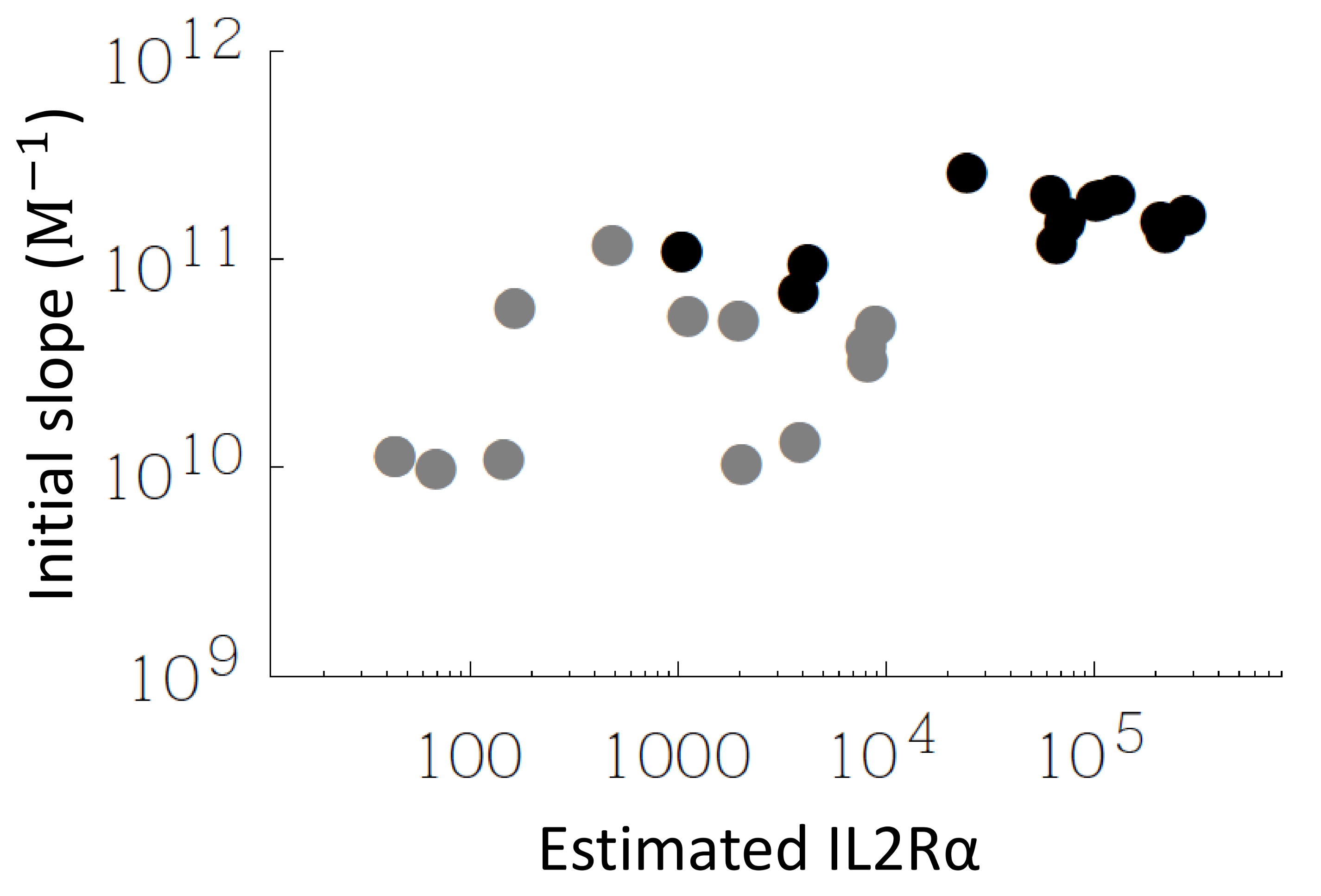

Supplement: S1 Fig — (TIF) [file pone.0155684.s004.tif]

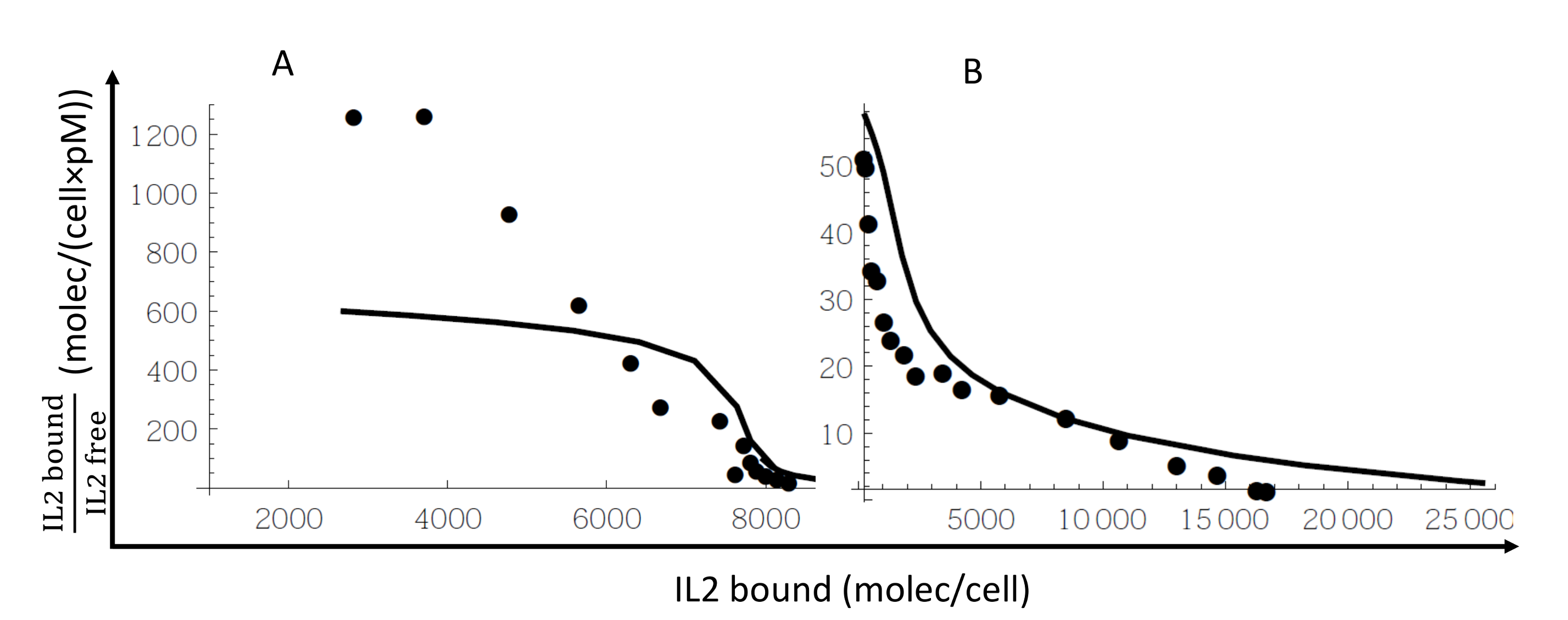

Supplement: S2 Fig — (TIF) [file pone.0155684.s005.tif]

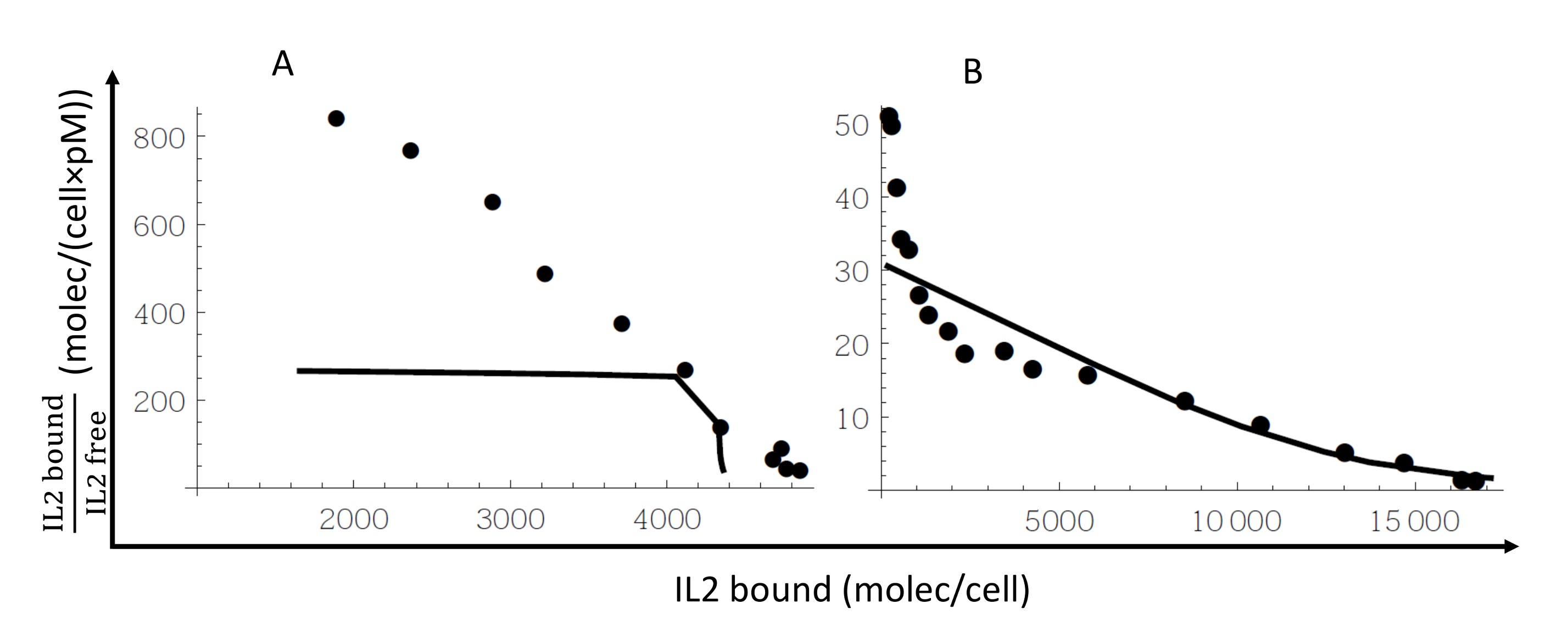

Supplement: S3 Fig — (TIF) [file pone.0155684.s006.tif]

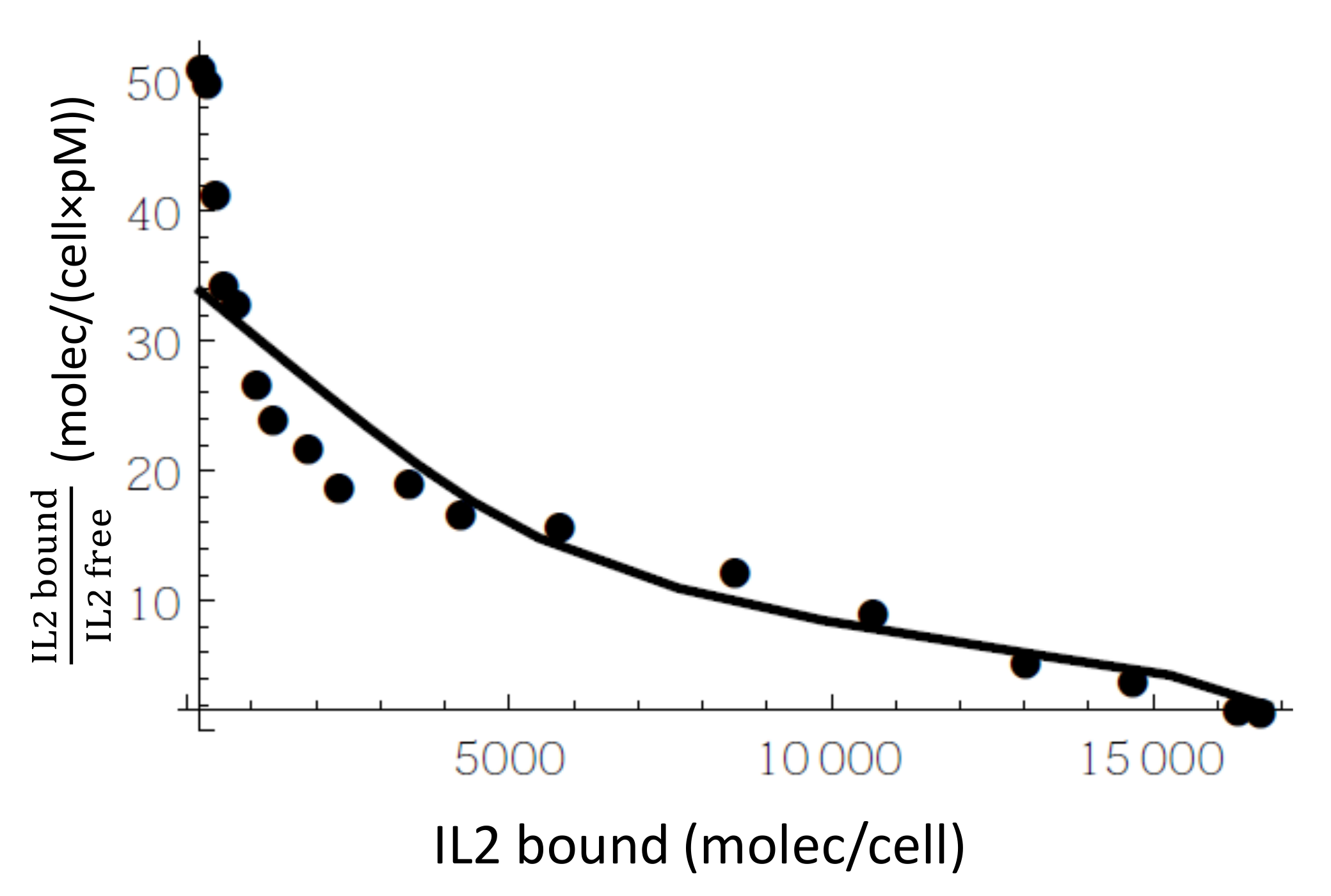

Supplement: S4 Fig — (TIF) [file pone.0155684.s007.tif]

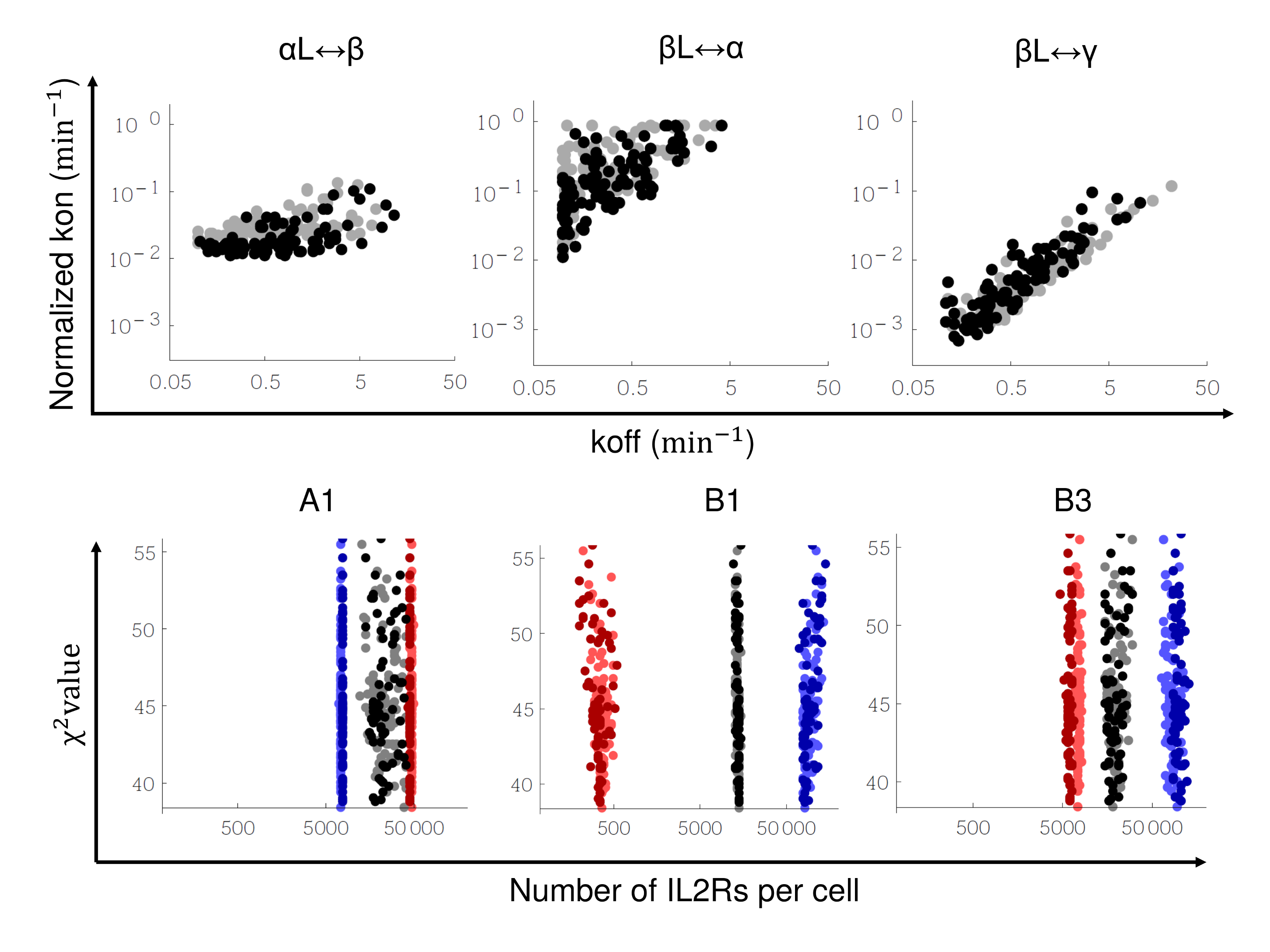

Supplement: S5 Fig — (TIF) [file pone.0155684.s008.tif]

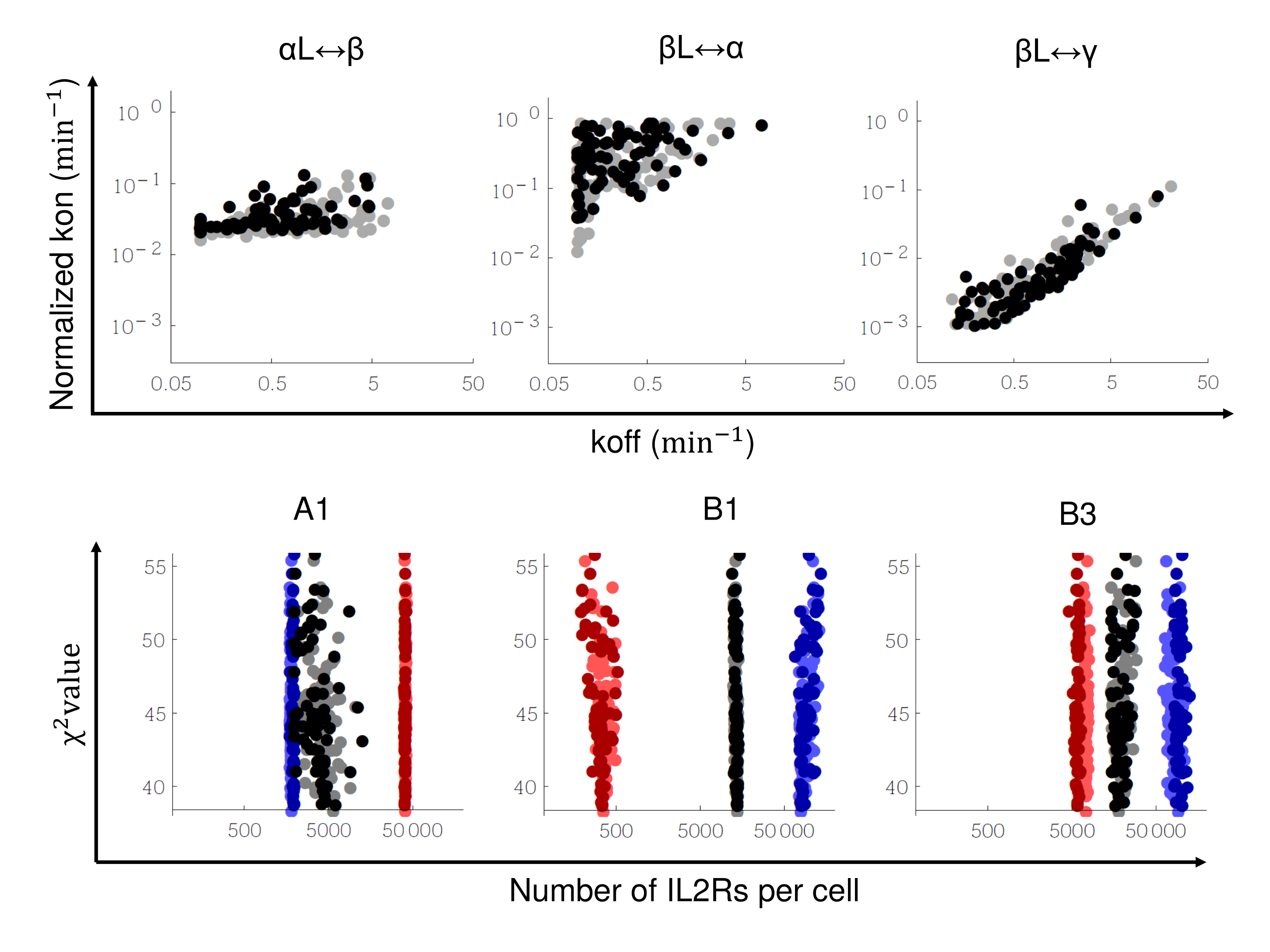

Supplement: S6 Fig — (TIF) [file pone.0155684.s009.tif]
